# To lockdown or not to lockdown: Analysis of the EU lockdown performance vs. COVID-19 outbreak

**DOI:** 10.3389/fmedt.2022.981620

**Published:** 2022-10-21

**Authors:** Emanuele Lindo Secco, Stefano Conte

**Affiliations:** ^1^ School of Mathematics, Computer Science and Engineering, Liverpool Hope University, Liverpool, United Kingdom; ^2^ Resiliency Services, IBM, Milano, Italy

**Keywords:** JHU coronavirus, Google mobility data, Apple mobility data, COVID-19 outbreak, national lockdown, national policies

## Abstract

The worldwide COVID-19 outbreak has dramatically called for appropriate responses from governments. Scientists estimated both the basic reproduction number and the lethality of the virus. The former one depends on several factors (environment and social behavior, virus characteristics, removal rate). In the absence of specific treatments (vaccine, drugs) for COVID-19 there was a limited capability to control the likelihood of transmission or the recovery rate. Therefore, to limit the expected exponential spread of the disease and to reduce its consequences, most national authorities have adopted containment strategies that are mostly focused on social distancing measures. In this context, we performed an analysis of the effects of government lockdown policies in 5 European Countries (France, Germany, Italy, Spain, United Kingdom). We used phone mobility data, published by Apple Inc. and Google, as an indirect measure of social distancing over time since we believe they represent a good approximation of actual changes in social behaviors. (i) The responsiveness of the governments in taking decisions. (ii) The coherence of the lockdown policy with changes in mobility data. (iii) The lockdown implementation performance in each country. (iv) The effects of social distancing on the epidemic evolution. These data were first analyzed in relation with the evolution of political recommendations and directives to both assess (i) responsiveness of governments in taking decisions and (ii) the implementation performance in each country. Subsequently, we used data made available by John Hopkins University in the attempt to compare changes in people behaviors with the evolution of COVID-19 epidemic (confirmed cases, new and cumulative) in each country in scope. Finally, we made an attempt to identify some key lockdown performance parameters in order to: (i) establish responsiveness, efficiency and effectiveness of the lockdown measures. (ii) model the latency occurring between the changes in social behaviors and the changes in growth rate of the disease.

## 1. Introduction

The spread of Covid-19 around the world in 2020 has called for appropriate responses from the governments. Following the early stages of the Covid-19 virus outbreak, scientists were able to estimate both the basic reproduction number and the lethality of this aggressive and infective disease: that made very clear the level of risk that this virus represents.

It is well known in the literature that the basic virus reproduction number depends on several factors such as

(i) the environment and the social behaviors (i.e., the probability that infected people get in contact with susceptible people)
(ii) the specific virus characteristics (i.e., the likelihood of transmission between an infected and a susceptible if they get in contact)
(iii) the removal rate, that is the rate at which infected people recover or die (assuming that recovered people becomes immune).

In the absence of specific treatments, such is the case of Covid-19 at today, there is a limited capability to control the likelihood of the transmission (i.e., vaccine) or the recovery rate (i.e., specific drugs). Therefore, in order to control the expected spread of the disease—which in the early stages of the infection typically mimics an exponential law—and to contain its consequences, most governments and national authorities have adopted a containment strategy, namely a lockdown policy.

This strategy—aimed at controlling the growth rate—is mainly focused on social distancing measures: the less are the contacts among individuals, the lower is the probability that an increasing number of people will catch the virus.

Of course, governments have complemented social distancing policies with other appropriate containment measures; they suggested or mandated the use of personal protection equipment (i.e., wearing masks in public areas) and promoted the increase in hygienic habits (i.e., frequent and accurate hands washing).

Nevertheless, we believe the lockdown policies play a key primary role to succeed in the control of coronavirus pandemic. Hence, we decided to investigate on the way they have been adopted and implemented around the world.

To the best of our knowledge, the research documented in this paper represents the first attempt to mix a set of interdisciplinary measurements of the COVID-19 outbreak.

Here we matched the actual population behavior vs. the government lockdown policies for 5 European countries (Italy, Germany, Spain, France and United Kingdom) which represents a significant amount of people vs. the overall European population. We will analyze the mobility trends of the national population in each country, with an inter-country comparison focusing on the derivative of such lockdown, namely the temporal decrement of the national population mobility vs. the lockdown as declared and imposed by local authorities (1, 2).

Due to the complex dynamics of the COVID outbreak there are clearly many aspects that affect its evolution that are not (yet) incorporated in this study, such as the role of hospitals and the effect of the quarantine measure vs. the spread of the virus (3, 4). These aspects are not taken into account in this study, even if they are important especially from an epidemic viewpoint.

In this context it is also important to emphasize that the analysis we are going to perform will specifically focus on the mobility data combined with the overall number of the compartments, i.e., the “metrics” of the infected individuals, recovered individuals and so on. We are not embedding within our analysis other relevant information which would enhance a better understanding of the outbreak dynamics from a more insightful medical viewpoint, namely from an epidemiological perspective (5, 6). For example, the role of hospitalization, the effect of vaccine and the distribution of the hospitals and of the poles of attractions are not examined here (7–9).

We will then look at the efficiency and efficacy of such mobility decrement with respect to the number of cases of infection in each country and will investigate the possible relationship between the implementation of lockdown and containment measures and the effects in the reduction of the epidemic.

Finally, we will try to assess the influence of the initial lockdown conditions (in terms of number of infected individuals) with the changes over time in the transmission rate of the disease. In other terms we will investigate to what extent the initial conditions influence the speed of reduction of the transmission rate.

Section 2 outlines the sources of data that we have used and some concerns about the possible entropy and quality of these data vs. the proposed analysis. Section 3 presents the pre-processing of the data, that is the data preparation and visualization. Section 4 is about the analysis and the results of these analysis. Discussion and conclusion follow in Sections 5 and 6, respectively. Following the References (Section 7), we also reported an Appendix or Section 8 where all plots for each country can be found.

## 2. A multi-disciplinary approach

This research investigates on the outcomes of lockdown policies put in place by national governments in 5 European countries.

The inquiry has been conducted on three key areas of investigation:

• a timeline of events has been developed to illustrate main facts and decisions related to the lockdown policies adopted by national authorities
• mobility data have been analyzed to investigate the effects of governments decisions on social distancing
• mobility figures have been compared to COVID-19 trends to grasp the effects of social distancing on the spread of the disease

This analysis makes use of three types of data:

• **Government and National Lockdown Timeline**—Lockdown timelines have been created using publicly available data mostly collected from the Internet. The intent here was to capture a concise representation of main facts and decisions that are related to the social separation policies adopted by governments.
  Comparison among lockdown policies is very complex and goes beyond the objectives of the present research. Some considerations about such complexity have been reported in par. 4.2.
• **COVID-19 data**—The World Health Organization (WHO) publishes daily coronavirus disease situation reports and provides data and information on the ongoing pandemic. Other efforts are put in place by several public and private organizations to gather, organize, aggregate and analyze data. As an example, John Hopkins University Centre for Systems Science and Engineering (JHU CSSE or JHU) is collecting data from several sources and makes them available to third parties.
  This research is based on data gathered and made available “to the public strictly for educational and academic research purposes” in the repository (10) by the John Hopkins University (JHU) CSSE (11). The datasets provide information about the spread of coronavirus in several countries around the globe in terms of confirmed cases, deaths and recovered patients.
• **Mobility Data**—Recently (April 2020) Apple and Google have released worldwide data of the mobility distribution of mobile phones for each country, i.e., data related to the mobility of all iPhones and Android Phones around the world.

We have processed all these data in order to look for possible links towards the COVID-19 epidemic and to characterize the effect of the government lockdown policy.

At this stage of the analysis, the data of 5 European countries have been analyzed, namely the data of France, Germany, Italy, Spain and United Kingdom. These countries were chosen because of their similarities from a geographical and cultural viewpoint, which makes a comparison easier. Moreover, all of them experienced the COVID or Coronavirus outbreak in a similar period and represent a large share of the overall number of COVID-19 cases in the European continent.

All analyses have been performed by developing code with the Python Programming language^1^ in the Jupyter Notebooks environment, a document format based on JSON^2^.

### 2.1. Data sources

In this paragraph we will focus on the sources we used to collect the three main categories of data needed to conduct the proposed analysis: (i) Lockdown data, (ii) COVID-19 data, and (iii) Mobility data.

• **Lockdown data** consist in a collection of significative events related to the Coronavirus outbreak or to the political decisions taken to contain the spread of the disease. This information has been mostly collected from public sources—such as Internet, press, media—or by interviews with local contacts.
• **COVID-19 data** are time series data describing overall trends associated to the spread of the epidemic. Our main data source for this category is the JHU CSSE.
• **Mobility data** used in this research basically describe how much time people spend in different locations, or how much time they intend to spend in travels, and how these habits change over time. For this category, we have used data made publicly available from Apple and Google.

#### 2.1.1. Lockdown data

All 5 countries examined in this research have been adopting social distancing measures to contain the spread of COVID-19 disease. The intent of lockdown policies is to limit contacts among individuals and thus reduce the risk of infection. Therefore, governments have put in place temporary restrictions on mobility and people were invited (or even forced) to stay at home unless they had valid reasons to move.

With the term *lockdown data,* we refer to a set of major events related to lockdown policies that occurred in each country under analysis: we collected and recorded those measures, ordinances, and facts that were significant for the population of each country. Information was retrieved from several sources, such as official channels, governmental sites, press and communication media.

We have also consulted residents in the country in order to discriminate and identify events that even if may not be considered as official governmental acts, had a relevant impact and strongly influenced the behavior of the population; sometime press conferences have strongly affected the perception and the reactions of the population: we may refer, for example, to the conferences held in Germany by A. Merkel on March 11th and March 18th.

Accordingly, we collected evidences of the most significant dates of such government impositions and sketched them in a graphical timeline format.

At time of writing, for all countries in scope, the epidemic seems to have reached its peak and the number of active cases is finally decreasing as the actual reproductive number is less than one. As a consequence, national governments are carefully easing their lockdown policies: in most cases that would represent a first step toward a normalization of people lives. Therefore, we have also tracked some of the events associated to this second phase.

Lockdown data and information have been collected from different sources, including, for example, local and National information sources, portals collecting the evolution of the outbreak in the different countries (1), websites, and media coverage (12–14), as well as professional figures who are residents and are stably living and working in the different countries (see the acknowledgment).

Table 1 lists some information related to the lockdown policies that has been gathered for the United Kingdom.

**Table 1:** Lockdown data for United Kingdom—L01 is (15), L04 is (16), L07 is (17), L14 is (18).

Date	Event
29-01-2020	British Airways suspends flights to mainland China [L14]
28-02-2020	First Case
29-02-2020	Outbreak
03-03-2020	Emergency Cobra meeting. PM says an outbreak across UK is “likely” [L01]
06-03-2020	First Death [L04]
12-03-2020	The UK moves from the “contain phase” to the “delay phase” [L01][L04]
	Self-isolation for vulnerable people
13-02-2020	Chief scientific adviser Patrick Vallance suggests that the UK's goal is to achieve “herd immunity” [L01]
	Downing Street says mass gathering will be banned from following week [L01]
15-03-2020	Plan to isolate elderly people [L04]
16-03-2020	First of Downing Street daily press conferences; BKJ advice to avoid all unnecessary contact and travels [L01]
17-03-2020	UK advises against nonessential travels abroad [L01]
20-03-2020	Schools Shut. Cafes, Pubs and Restaurants Close [L01]
	Cafes, pubs, and bars to close, as well as shops, theatres and leisure centres, are to close to protect public health. [L04]
22-03-2020	BJ warns he could have to introduce tougher measures [L01]
27-03-2020	BJ is diagnosed with the virus [L07]
05-04-2020	Queen Elizabeth calls for “self-discipline and resolve” to defeat the coronavirus. [L01]
09-04-2020	Dominic Raab signals that the UK lockdown will be extended [L01]
23-04-2020	Nationwide Lockdown
10-05-2020	Start of Phase 2

#### 2.1.2. COVID-19 data

The World Health Organization (WHO) publishes daily coronavirus disease situation reports and provides data and information on the ongoing pandemic. Other efforts are put in place by several public and private organizations to gather, organize, aggregate and analyze data.

This research is mostly based on data gathered and made available “*to the public strictly for educational and academic research purposes”* by JHU in a GitHub repository (10, 11). The JHU CSSE, in turn, is collecting and organizing data coming from several primary sources. The resulting dataset provides information about the spread of coronavirus in several countries around the globe in terms of *confirmed and active cases, deaths and recovered patients*.

In fact, according to the definition reported in (11), 4 compartments are reported for each country on a daily basis, namely:

• ***Confirmed:*** the total number of cases recorded by each country up to each day (this is a cumulative value); this number sometime includes the presumptive positive cases and the probable cases.
• ***Deaths*:** this number accounts for confirmed and—for some countries—probable deaths due to coronavirus illness. Deaths are included in the Confirmed cases.
• ***Recovered*:** this value is an estimate of the number of individuals who have recovered from the disease and is determined “based on local media reports, and state and local reporting when available, and therefore may be substantially lower than the true number”. Recovered are included in the Confirmed cases.
• ***Active*:** represents the total number of people that result as infected at a given date, namely Confirmed cases less the Recovered cases, less the Deaths.

**Figure 1:**
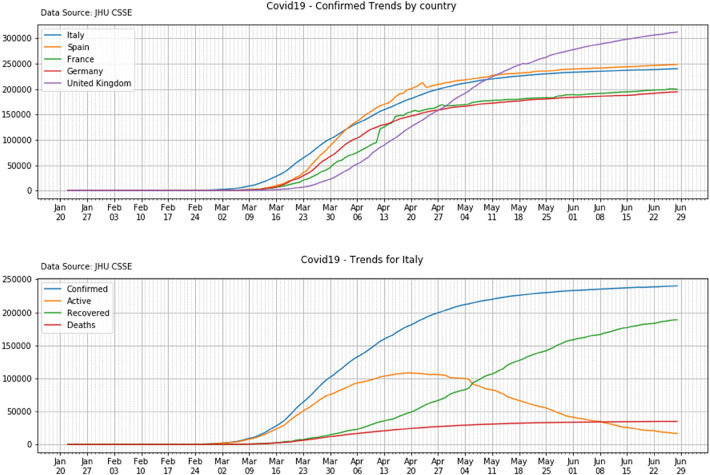
Top panel: the trends of the confirmed cases for the five countries in scope. Bottom panel: the Confirmed, Active, Recovered and Deaths values for a single country: Italy.

Figure 1 (top panel) shows the trends of the Confirmed cases of the 5 countries under investigation. The bottom panel plots all the different compartments for a single country, Italy.

#### 2.1.3. Mobility data

In April 2020, Apple and Google have publicly released sanitized data describing changes in mobility trends of individuals for several countries around the world (19, 20). These data actually track different phenomena (request for directions vs. presence of people in specific locations) but, indirectly, provide a measure of people mobility over time.

##### 2.1.3.1. Apple mobility data

Apple has reported all data reflecting “requests for directions in Apple Maps” in (19). The company clarifies that, “this data is generated by counting the *number of requests made to Apple Maps* for directions in select countries/regions, sub-regions and cities” and “the availability of data in a particular country/region, sub-region or city is based on a number of factors, including minimum thresholds for direction requests per day” (19).

According to such a definition, these data do not represent the effective movements of the end-user, rather they represent the interest or intention of the user to reach a certain destination by different means, namely walking*,* driving or transit.

Apple data describe changes in mobility in percentual terms with respect to a predetermined *baseline reference*. This initial reference level (100%) has been defined as “a baseline volume on January 13th, 2020”.

**Figure 2:**
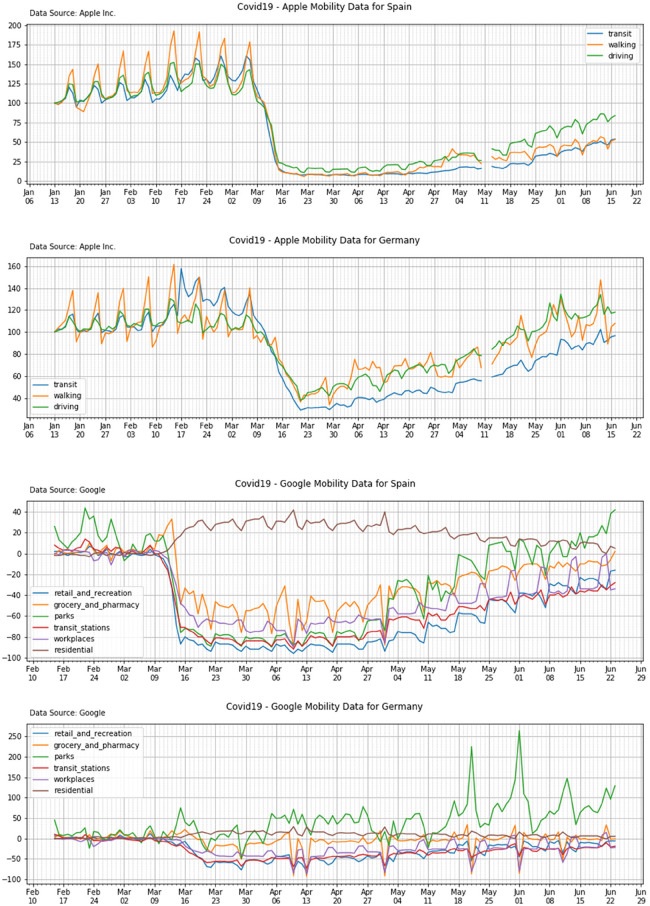
Apple mobility trends (top panels) and Google mobility trends (bottom panels) for Spain and Germany.

Figure 2 (top panel) shows Apple mobility trends for two among the countries in scope: Spain and Germany. Percentual changes in transit, walking and driving (y-axis) are reported as function of time (dates on x-axis). Percentual changes are expressed with respect to the baseline reference (100%) as defined by Apple.

##### 2.1.3.2. Google mobility data

Google has publicly released the COVID-19 Community Mobility Reports (COVID-19 Community Mobility Report, 2020), namely a set of data collected by Google OS from the available mobile phones around the globe.

Google dataset is conceptually different from the Apple dataset. “Each Community Mobility Report dataset is presented by location and highlights the percent change in visits to places like grocery stores and parks within a geographic area” (20). Google data are classified into 6 categories and they represent *the effective location of the end-user* mobile phone. The categories are (1) *Retail and recreation, (2) Grocery and pharmacy, (3) Parks, (4) Transit stations, (5) Workplaces and (6) Residential.*

In order to provide a baseline reference for these data, the Google “datasets show how visits and length of stay at different places change compared to a baseline” and “the baseline is the median value, for the corresponding day of the week, during the 5-week period Jan 3–Feb 6, 2020”.

Figure 2 (bottom panel) shows Google mobility trends for two among the countries in scope: Spain and Germany. Percentual changes in average time spent in each category of locations are reported (y-axis) as a function of time (dates on x-axis). Percentual changes are expressed with respect to the baseline reference (0%) as per Google definition.

### 2.2. Data quality

In this paragraph we will share some considerations on the quality of the data we have been using in our research. Before performing any analysis, it is important to focus on the nature, variability and noisiness of these data to ensure they fit the purpose.

We are fully aware that the data from all three categories are noisy and with a limited level of accuracy. That's not a surprise and there are several reasons for this. For example, we know that COVID-19 data come from a variety of sources and from different countries with different criteria for data collection and different delays in reporting times.

When we are aware of the level of accuracy of our data, we can still analyze and compare them to draw some conclusions, as long as we can accept some degree of uncertainty.

#### 2.2.1. Lockdown data

Depending on country specific situation, political decisions on social distancing measures have been changing over time. In some cases, restrictions were applied locally (where COVID-19 clusters were initially detected) and were later extended to larger areas of the country up to the national level. Moreover, permitted activities during the lockdown phase vary country by country: this makes the analysis of such policies and their comparison really complex, but an in-depth analysis of lockdown policies is not an objective of this research (1).

According to our objectives, we have been collecting data on what we considered the “main” and “significant” events for each country. We took into account the official announcements of lockdown measures (implemented at a regional or national level) as well as all those facts that had a strong influence on the public perception of the COVID-19 outbreak and, therefore, on the population's behavior.

Lockdown information we have used are somewhat “qualitative” in nature: as we said, they are intended to describe main events and decisions that are related to the development of the epidemic or to the evolution of the social distancing regulations imposed by the national authorities. As a matter of fact, our data collection is undermined by some intrinsic weaknesses; let us briefly discuss some of them.

##### 2.2.1.1. Arbitrariness and completeness

Our selection of “relevant events” is obviously arbitrary and subjective: other choices could have been equally good, or even better. The collection is by no means exhaustive: for sure, some of these events may have been missed, even if the authors have tried to recover all sets of these main events through the references to different media channels and the direct contact with residents in the different countries.

##### 2.2.1.2. Same term, different meanings

We are aware that the same term, for example “Nationwide Lockdown”, has “more or less” the same meaning everywhere, but—in facts—it does not indicate exactly the same thing in all countries under observation since the social distancing measures and the way restrictions have been implemented differs from country to country.

##### 2.2.1.3. Time consistency

Sometime data sources do not fully agree on dates: maybe in some cases one data source records when a political decision has been taken while another refers to the date when that political decision became effective. Sometime things are unclear.

##### 2.2.1.4. Complexity

Simple tasks are not always easy. For example, we wanted to identify the day that marks the beginning of the epidemic in each country: we considered that a fundamental milestone. The attempt was not as straightforward as we would have initially expected:

• First COVID-19 cases in Italy were recorded from Chinese tourists visiting the country. The infection, very likely, was not contracted in Italy: so, in our opinion, that event is not a proof that the disease was spreading in the country. Therefore, for all countries in scope, we have been looking for cases with evidences of local transmission.
• Germany had a local cluster of Coronavirus cases in January, with confirmed local transmission, but that cluster was associated to a specific situation that occurred in Bavaria. The cluster has been kept under control for weeks—about 15 people being involved in total—and only much later (at the end of February) Germany experienced its COVID-19 outbreak.

So, even the “first case” with confirmed transmission within a country does not always mark the beginning of the epidemic spread within a nation. Therefore, we had to use a proper and reasonable judgement to identify the “first case” that was relevant for us, the one that marks the outbreak of COVID-19 within the country.

#### 2.2.2. COVID-19 data

For sure all efforts are done to provide correct information, but it is well understood that the accuracy of available data cannot be given for granted. JHU itself warns the user of its dataset on the fact that that its “website relies upon publicly available data from multiple sources, that do not always agree” and clarifies that “confirmed cases include presumptive positive cases”. We are also informed that “recovered cases outside China are estimates based on local media reports, and state and local reporting when available, and therefore may be substantially lower than the true number” and that “death totals in the US include confirmed and probable”.

Other clues suggest that data signals are noisy, and this aspect should be taken into account while performing our analysis. It is important to clarify some of these clues.

##### 2.2.2.1. CFR as an indicator of lethality

A quick at look at the available data will show very different Case Fatality Rates (CFRs) in different countries. CFR is determined as the ratio between COVID-19 deaths and confirmed cases and its accuracy depends on the accuracy of those values. It has been observed that “by 24 March 2020, Italy's case fatality rate (CFR) was nearing 10%, while China's hovered at around 4% and Germany recorded a much lower figure, at 0,5%” (Villa, 2020).

Of course there may be several causes that determine those figures such as a different age distribution in the population or a different quality of healthcare system, but such factors do not fully explain the big differences in countries like Italy and Germany that are not too far, both in terms of geography and social development.

Some observers have suggested that the primary reason should be given by the fact that confirmed cases just represent a subset of all active infections, that is “the tip of the iceberg”. The share of known cases may differ by a great extent from country to country since countries have different capabilities and policies in place to perform the screening of the population. Moreover, it has been observed that both policies and capabilities in a given country have been changing over time.

##### 2.2.2.2. Recovery times

The first cluster of coronavirus cases in western countries took place in Italy starting on February the 20th, 2020. Starting from that day Italy experienced an exponential growth of confirmed infections. The outbreak in Spain started a few days later and had an initial lower rate of growth. A couple of weeks later the growth rate boosted in Spain until, on April 4th, the number of confirmed cases reported from Spanish authorities was higher than the Italian figure.

Since the Italian cluster started earlier and the number of confirmed cases in Italy has been much higher than the Spanish one for several days, we would have expected a similar behavior for the number of the recovered patients. That is not the case: it looks like recovery times are much shorter in Spain. This is what we may conclude looking at this statistic, based on data collected on April 17th, where the number of individuals who have recovered in Spain almost doubles the number of recoveries in Italy. Is Spanish healthcare system better than the Italian ones? If so, why are deaths statistics almost parallel?

Again, chances are that we formally have the same data from different countries, but there are different procedures, criteria and timings to count recovered individuals.

##### 2.2.2.3. Mortality and COVID-19 lethality

The Italian National Institute of Statistics (ISTAT) has recently made available mortality data referring to the period January the 1st—April the 4th of years 2015–2020. The initial period of 2020 shows a significant increase in mortality if it is compared with the same period in the previous years. Such a difference is much larger than the total number of deaths which have been reported in the COVID-19 statistics as provided by Italian authorities. Again, chances are that we formally have the “same” data from different countries, but the meaning, criteria and timings of these data may be different.

#### 2.2.3. Mobility data

As we said, our intent is to use mobility data as *an indirect measure of social distancing*: as people mobility decrease, lower are the chances that people get in contact. Lower social contacts, in turns, mean a lower risk for individuals to get infected by Coronavirus.

Apple data are an expression of a potential interest to navigate—by different means—to a certain destination, while Google data represent the time spent by individuals' mobile phones in locations belonging to a given set of categories.

It is difficult to determine how accurate these indirect measures are for our purposes. But, given the different meaning and nature of Apple and Google datasets, we decided to compare these data for each country under analysis in order to verify if—at least—they are consistent in term of trends.

In fact, similar trends from these different datasets would suggest that in some way they provide a fair estimate of changes in social contacts among time, in other words an agreement in trends would suggest that there is some fair degree of correlation among the quantities measured by Apple and Google and the quantity we want to estimate indirectly.

**Figure 3:**
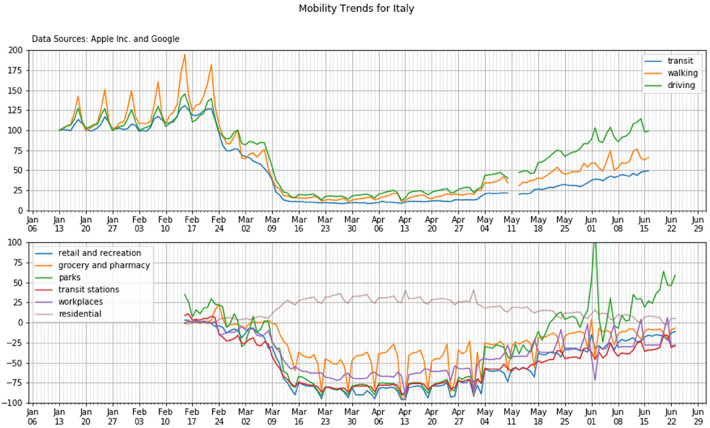
Time alignment of the Apple and Google data on the top and bottom panels, respectively (country: Italy).

Figure 3 shows this comparison for Italy. In the figure we have marked some significant events such as the activation of the government lockdown (see par. 3.1.1), which are represented with a set of red vertical lines. In order to well represent the timeline of the event, we also clustered the data week by week reporting a set of vertical grids made of groups of 7 days each (light grey vertical lines in the figure).

The figure shows a first order of consistency, since all Apple time patterns decreased at the lockdown events and, simultaneously, the residential Google data show a clear increment; at the same time all other categories of the Google data display a reduction. Both Apple and Google data exhibit a weekly pattern with some variations in the weekend.

Moreover—even if a deeper analysis would be desirable—such consistency suggests that the intentional Apple data may effectively represent the consequent localization of the end-user.

## 3. Data preparation and visualization

cIn Section 2 we have clarified the sources and nature of the data sets that we are going to use for our analysis. At this stage we are now going to define a set of process and parameters on which our analysis will be based on. For clarity, we divided this part into different subpar. focusing on each data type.

### 3.1. Lockdown data

In order to process Lockdown data according to our objectives, we split our observation time of the epidemic and of the consequent political decisions into 3 different stages:

• ***STAGE 0*:** starting immediately after the COVID-19 outbreak in each country and ending when restrictive policies imposed by local authorities reach their maximum;
• ***STAGE 1*:** during the nationwide lockdown period in each country, when most social activities are banned or restricted to minimize the risk of transmission of the disease;
• ***STAGE 2*:** beginning when national governments progressively start easing restrictions on people activities;
• ***STAGE 3*:** starting when people are finally permitted all usual social activities, even if more stringent regulations are imposed to carry on the activity (i.e., wearing masks)

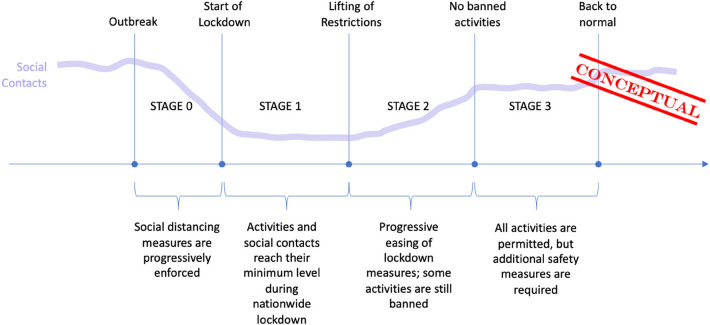


This research mostly focuses on the initial stages; therefore, we have gathered a partial list of key decisions and events associated to the evolution of the lockdown policies.

We have defined two classes of events:

• ***Key events*** that are “common” to all the 5 countries under analysis; they mark major facts and usually represent a change of stage, such as the First Case recorded (COVID-19 Outbreak) or the beginning of the Nationwide Lockdown.
• ***Relevant events*** may be common to all countries (shut of schools and universities) or specific of a given nation; for example, we collected facts that provide clues about the evolution of the lockdown policies during STAGE 0 (i.e., the time period between the COVID-19 Outbreak and the imposition of the Nationwide Lockdown).

#### 3.1.1. Key events

##### 3.1.1.1. Outbreak

In our view, the Outbreak is the key event that marks the beginning of STAGE 0. It represents what we believe to be the “first case”, that is the COVID-19 case that triggers the spread of the epidemic disease within a country. Be aware that this is not always the first case recorded within the country, nor the first case actually contracted within the boundaries of a country.

In order to determine the Outbreak date for each country in scope, we had to consider several events from the lockdown data and also compare them with other parameters (i.e., the number of confirmed COVID-19 cases).

• **First Case:** date of first local case (14), namely the detection of an infection developed within the country boundaries. Positive cases from visitors (i.e., people arriving and tested positive in the country because they were infected elsewhere do not prove that the infection is active within the country).
• **First Cluster:** In some situations, a single illness case—immediately detected and isolated—may not trigger an epidemic. In epidemiology “a cluster is an aggregation of cases of a disease or another health-related condition […] closely grouped in time and place” (21).
• **Outbreak:** a local small cluster does not always imply an immediate spread of the infection within a country. If the cluster is local and under control, it has been happening in Germany for Covid-19, we can't always say the epidemic is started. So, we tried to define the Outbreak as the date when a significative number of cases have been recorded, followed by exponential increase.

##### 3.1.1.2. Start of lockdown

The Start of Lockdown is the *key event* that marks the transition between STAGE 0 and STAGE 1 of our observation period. For each country under analysis, it represents the first day of the Nationwide Lockdown imposed by the political authorities.

For us, the term Nationwide Lockdown represents the time period when all non-essential activities are banned, and people are mandated to stay at home and avoid social contacts.

In some cases, i.e., Italy, the rules defined by a government for the Nationwide Lockdown have changed over time. Therefore, in order to determine the Start of Lockdown, we decided to consider the day when restrictive policies, in each country, have reached their maximum level.

##### 3.1.1.3. Lifting of restrictions

According to our definition, STAGE 2 begins when national authorities start easing restrictive measures. In fact, at time of writing, all countries in scope have moved to a “next phase” when a gradual re-start of business and social activities is being allowed by governments.

#### 3.1.2. Relevant events

Relevant events have been collected and used in our research to help identify key events and to analyze potential linkages with other data. In fact, as part of the present research main facts and decision related to the lockdown policies have been gathered to be compared with data of different nature such as people mobility data and data describing the evolution of the coronavirus disease.

#### 3.1.3. Processing / representation

Most of processing for lockdown data was manual: information was gathered, selected and organized in table format for human analysis. Table 2 reports some of the main events that have been recorded for each country under analysis.

**Table 2:** Summary of the main relevant lockdown milestones of the 5 countries.

2020 Lockdown Milestone				
Country	First Case	Significant Dates / Events	National Lockdown	Ease of Lockdown
France	25/02	29/02—epidemic stage 2	17/03	11/04
		14/03—epidemic stage 3		
Germany	27/01	11/03—social distance alert (conf press 1)	22/03	15/04
		18/03—pandemic status (conf press 2)		
Italy	20/02	21/02—lockdown of Province of Lodi	09/03	04/05
		04/03—schools/universities closure	11/03	
		08/03—lockdown of Northern Provinces	lockdown tightening	
Spain	24/02	13/03—state of alarm	15/03	13/04—lift on restriction
				02/05—de-escalation
United Kingdom	29/02	12/03—self-isolation for vulnerable people	23/04	10/05
		20/03—closure of schools, pubs, restaurants		

In this context, the most important and significant dates of each country lockdown have been considered and reported in Table 2 (1–14). This table not only reports the beginning of the national lockdown, but also refer to other significant dates which have influenced the population behavior of each country: for example, when referring to Germany, two dates of conference press of the prime minster have been reported since these conference press have deeply influenced the perception and behavior of the population vs. the COVID-19 outbreak.

Lockdown data have also been represented through a graphical timeline, to be plotted together with COVID-19 or mobility data, and to produce holistic charts. Since we have been using Python Programming Language^1^ and Python libraries, such as Matplotlib, to accomplish this, we used basic Python data structures (i.e., dictionaries) to represent these data.

### 3.2. COVID-19 data

In order to perform the analysis and process all types of information together, we must perform some data preparation and pre-processing of the COVID-19 data from JHU CSSE, precisely:

• we have filtered the COVID-19 worldwide original data in order to extract the subset referring to the 5 countries in scope
• starting from these original 5 subsets we have identified the Confirmed, Active, Recovered and Deaths cases of each country and then defined 4 homologous “new” compartments—namely the *NewConfirmed, NewActive, NewRecovered* and *NewDeaths* cases—that represent the daily change (differential or derivative) of each original metric, respectively.

The definitions of these new compartments follow.

#### Derivative of confirmed, active, recovered and deaths

Here we report the calculation of the *NewConfirmed* cases, however the differential is calculated in the same way for each one of the other metric. If *C(t)* represent the *Confirmed* time series*,* then the *NewConfirmed* metric*, NC (t),* at the time or date *t_n_* is computed as:

$$NC(t_{n})=C(t_{n})-C(t_{n-1})$$

Therefore, it holds that:

• *NewConfirmed*, *NC(t)* represents—for each given day—the number of the new COVID-19 cases that have been confirmed vs. the cases of the previous day. This value should be always greater than zero or equal to zero. Unfortunately, this is not always true, possibly because errors in data were later amended in some of the countries’ databases.
• *NewActive*, *NA(t)*, represent—for each given day—the difference in active COVID-19 cases between that day and the previous one. This parameter can also assume negative values.
• *NewRecovered*, *NR(t)*, represent—for each given day—the number of patients that have been declared recovered between the day of the observation and the day before.
• *NewDeaths*, *ND(t)*, represent—for each given day—the number of infected individuals who have died for the COVID-19 disease between the day of the observation and the day before (i.e., in the last 24 h).

### 3.3. Mobility data

A set of procedures and parameters have been defined in order to process the mobility datasets and properly synchronize them with the other datasets (i.e., the lockdown data and the COVID-19 data).

#### 3.3.1. Apple mobility data

As we have already outlined, the Apple mobility data include three different metrics, namely the transit, walking and driving mobilities, respectively. In order to perform the analysis and conglomerate these data with the other information, we have defined a new unique and overall metric, or overall mobility, which is the average of the three-original metrics. We also used a smoothing filter to this average curve applying a 7-days rolling average to this newly defined metric.

The choice of a 7-days rolling average is clearly subjective—since other width of the time window maybe adopted—and may cause information loss, however it is reasonable to look at the weekly pattern in this context. Moreover, the intent here is to use the mobility data in order to detect possible changes of the people behavior—especially in terms of social distancing—before and after the COVID-19 outbreak.

Finally, as a result of the aforementioned processing of the data, we obtained a unique curve that (i) follows the trend of the original 3 metrics and (ii) highlights two main plateaus that are clearly related to the changes of the people behavior around the COVID-19 lockdown.

Here below, we detail the main steps of this process.

##### 3.3.1.1. Overall mobility trend

The 3 Apple mobility trends timeline—i.e., transit, walking and driving—have averaged according to the following expression:

$$A_{m}(t)=[M_{t}(t)+M_{w}(t)+M_{d}(t)]/3$$

where *A_m_ (t)* is the timeline of the average mobility and *M_t_ (t)*, *M_w_ (t)*, *M_d_ (t)* are the transit, walking and driving mobility trends timeline, respectively.

##### 3.3.1.2. 7-days Rolling average

The overall mobility *A_m_ (t)* is then filtered with rolling window calculation to obtain the mobile average of the 7-days average mobility, according to the following expression:

$$\begin{array}{rl} S_{m}(t)= & \;[A_{m}(t-3)+A_{m}(t-2)+\cdots +A_{m}(t+2)\\ & +A_{m}(t+3)]/7 \end{array}$$

where *S_m_ (t)* is the final 7-days rolling average.

##### 3.3.1.3. Differential of the mobility data

As we already mentioned, two plateaus can be easily identified in the 7-days rolling average mobility curve that we derived, namely

• an *upper plateau* representing the baseline or “usual” behaviour in terms of people mobility before the COVID-19 outbreak
• a *lower plateau* representing a steady state of reduced mobility occurring after the social distancing measure has become effective

**Figure 4:**
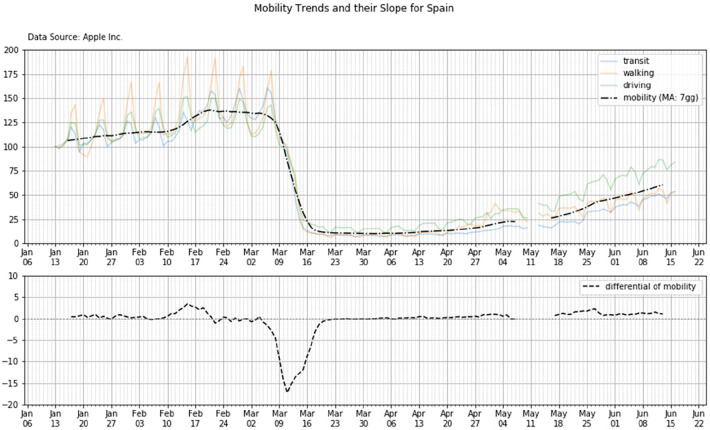
Top panel—the 7-days rolling average mobility as extracted from the original data set provided by Apple: the residential mobility trends are averaged and then smoothed (black dotted line). Bottom panel—the derivative of the 7-days rolling average curve—Country: Spain.

The 7-days average mobility plot also can help to characterize what happened in each country during the transition period between the two plateaus (Figure 4)

To this aim, in order to compare the main characteristics of the mobility trends of the 5 countries, we calculated the differential or derivative of such mobility curve: the differential has been calculated assuming a unitary time interval of 1-day. Therefore, the derivative of the mobility represents the instantaneous or daily rate of change of the mobility function over time, namely the numerical difference between mobility values measured at time or date t and at time *t−*1.

Figure 4 shows the mobility trend (upper subplot) and its derivative (lower subplot) for the country Spain.

#### 3.3.2. Google mobility data

Multiple metrics are also reported within the COVID-19 Community Mobility Report data as released by Google. As we did for the Apple mobility data, we wanted to derive a unique overall metric to summarize all metrics information for each of the countries in scope.

As we have already observed, Apple and Google data measure phenomena that are somewhat related, but different in nature. Despite baselines, and percent changes with respect to the baselines, are used in both datasets, different criteria and methods were employed to calculate them.

We have seen that all three Apple metrics showed similar percent changes over time, so we decided to use a simple average to derive the overall mobility trend from those data. Things are quite different for data provided by Google. In facts, considering for example the data of the United Kingdom, it holds:

• all metrics but one (i.e., the residential) decrease over time: because of the enforcement of social distancing measures people started spending more time at home and less time elsewhere;
• all decreasing metrics from the Google dataset show different percentual changes before and after the COVID-19 spread: for example, about 20% less time is spent in grocery and pharmacy vs. 70% less time spent in transit stations.

Therefore, a simple mean to derive a summary mobility trend cannot be used: a weighted average to account for the different amounts of time spent by individuals in different locations (i.e., workplace vs. retail and recreation) may be used; unfortunately the process to define and assign a weight to each metric, even if it may be based on a good common sense, it would have been very subjective and arbitrary.

Finally, we opted for a different approach. We derived an outdoor metric based on the following consideration: when people are not at home (i.e., residential), for sure they are somewhere else (i.e., outdoor). Therefore the 24 h per day can be partitioned into two subsets: a residential time and an outdoor time.

##### 3.3.2.1. Overall mobility trend: outdoor

Let us assume that—because of the social distancing measures—people change their behavior and, on average, spend less time outdoor (for example 4 h): that timeframe moves from the outdoor to the residential share. From the Google data set we are then informed about this increase of time spent at home in terms of a percentual change; unfortunately, we are not informed about the absolute value of this time. We know that the percentual increase in residential corresponds to a decrease of time spent outdoor, but we cannot determine the percentual decrease since we do not know the initial split between residential and outdoor.

Therefore, we decided to express the change over time in the outdoor category as a percent with respect to the residential metric: in practice, we defined the outdoor metric, *O_m_* (*t*), as the opposite of the residential metric, *M_r_ (t)*:

$$O_{m}(t)=-M_{r}(t)$$

The result is that the percentual change is the same in absolute terms, but opposite in sign: we have a percent increase in residential and the same percent decrease in outdoor. |For clarity, it is important to notice that those percent values are different in nature vs. the Apple data percentage values and therefore refer to a different set of scales.

##### 3.3.2.2. Smoothed mobility trend: 7-days rolling average

The *O_m_ (t)* is then filtered with a rolling time window of 7-days to obtain a 7-days rolling average mobility, according to the following expression:

$$S_{m}(t)=\frac{\left[O_{m}(t-3)+O_{m}(t-2)+\cdots +O_{m}(t+2)+O_{m}(t+3)\right]}{7}$$

where *S_m_ (t)* is the smoothed curve.

##### 3.3.2.3. Differential of the mobility data

As we did for the Apple mobility, the differential or derivative of the Google mobility curve is calculated in order to analyze the main characteristics of the data trends and compare the time patterns of the five countries.

The differential is calculated assuming a unitary time interval of 1-day. Therefore, the derivative of the mobility represents the instantaneous or daily rate of change of the mobility function over time, namely the numerical difference between mobility values measured at time *t* and at time *t−1*.

## 4. Analysis and results

**Lockdown policies and changes in people behavior**

These data were first analyzed in relation with the evolution of political recommendations and directives to assess a multiple set of performances of the different countries, namely:

(i) The responsiveness of the governments in taking decisions
(ii) The coherence of the lockdown policy with changes in mobility data
(iii) The implementation performance in each country
(iv) The effects of social distancing on the epidemic evolution

### 4.1. Lockdown policies: responsiveness of the governments

Here we measure the responsiveness of each government towards the beginning of the COVID-19 outbreak with a specific attention to each national infection.

#### 4.1.1. Objective / methodology

At first instance we look at the status of the infection at specific temporal moments and, in particular, in correspondence with the government decisions: a first set of graphs have been designed where we show the *NewConfirmed* (definition in par. 3.2) vs. the main lockdown events.

To estimate the authorities' responsiveness, we measure a time-based parameter and an epidemic one. The first one is a measurement of the *time response* of the government vs. the detection of the outbreak whereas the latter one returns the size of the *severity* of the outbreak.

(i) *Time response*—This variable is the time-based parameter and it is defined by means of two time-based parameters
  • The date of the detection of the first case in the country (T_i_), where for first case we intend a subject with the citizenship of that country and not any guest and/or tourist and/or foreign subject
  • The official date of the lockdown (T_f_) as it was engaged at national level, where for national we intend a procedure overall the whole nation and not just a local or restricted regional lockdown

Then, it holds that the government *time response* (Δ*T*) is defined as:

$$\Delta T=T_{f}-T_{i}$$

(ii) *Severity—*This variable is the epidemic-based parameter and it represents the extent of the outbreak when the national lockdown is onset. It is defined as the number of by means of two epidemic-based parameter

#### 4.1.2. Results (country by country)

##### 4.1.2.1. France

**Figure 5:**
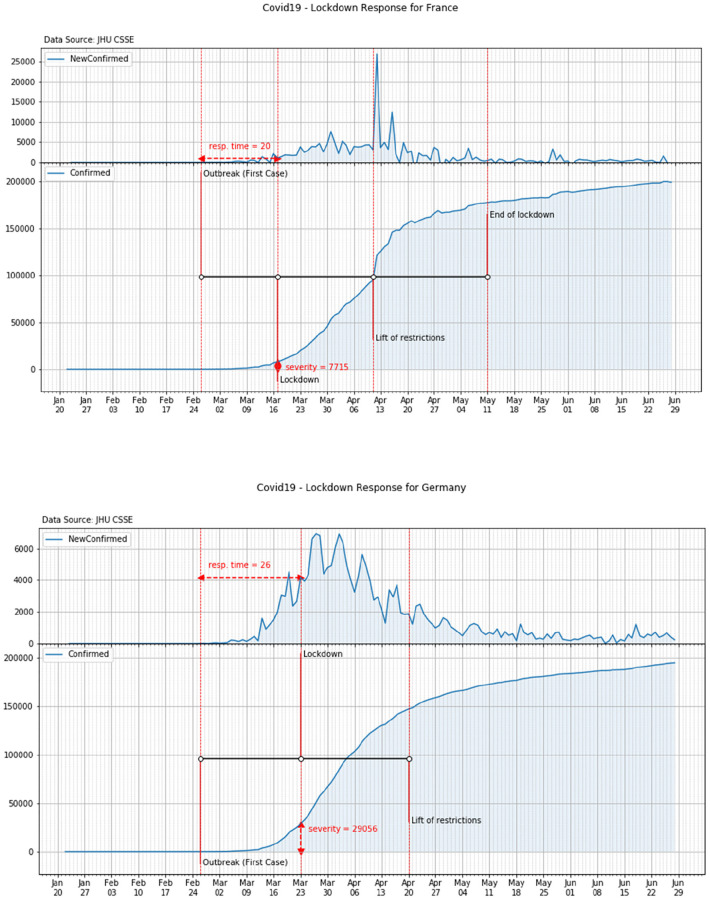
Response time and severity of the lockdown in France (top panel) and Germany (bottom panel).

French government formally imposed a nationwide lockdown on March 17th, 20 days after Coronavirus outbreak. Earlier some other restrictions—such as shut of schools and universities—were already put in place by national authorities. On March the 17th, according to JHU CSSE data, almost 8 thousand of the COVID-19 cases were confirmed in the country (Figure 5, top panel).

##### 4.1.2.2. Germany

As a consequence of the COVID-19 outbreak (February 26th), German authorities progressively established restrictions on social activities. Following the Prime Minister press conference, a tight nationwide lockdown was also established on March the 23rd, i.e., 19 days after the outbreak. On that day about 29 thousand of the COVID-19 cases were already confirmed in the country (Figure 5, bottom panel).

##### 4.1.2.3. Italy

**Figure 6:**
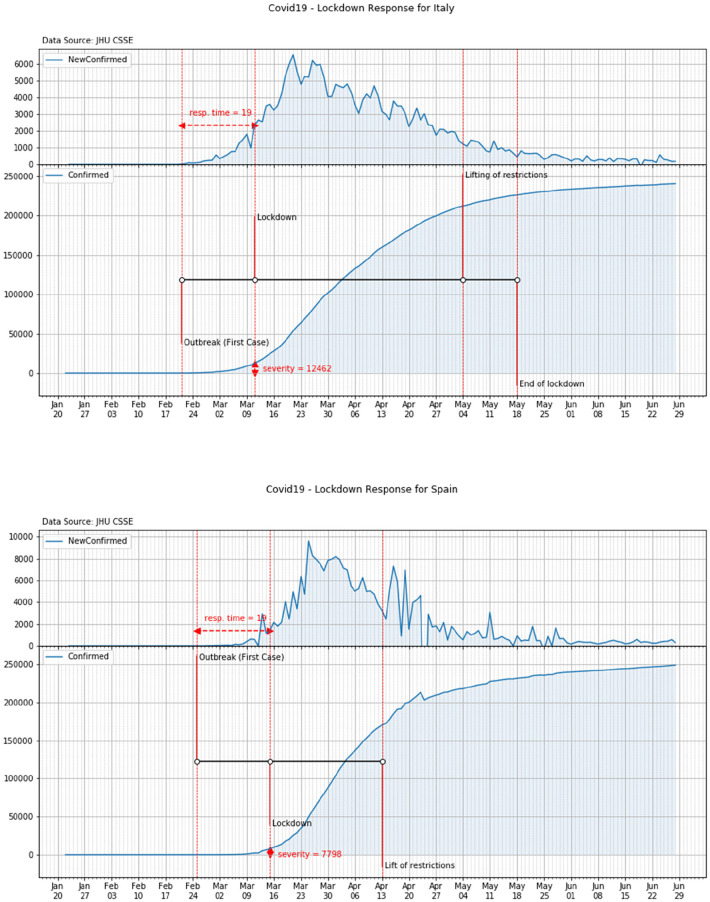
Response time and severity of the lockdown in Italy (top panel) and Spain (bottom panel).

Italy experienced COVID-19 outbreak on February the 21st. National authorities implemented immediate responses in the attempt to contain the spread of the disease. In the following days, as a consequence of the exponential growth of the cases, additional restrictions were gradually imposed by the government. Finally, on March the 11th a tight nationwide lockdown was mandated. On that day over 12 thousand of the COVID-19 cases were already recorded in the country (Figure 6, top panel).

##### 4.1.2.4. Spain

In Spain the outbreak of COVID-19 started on February the 25th when a citizen form Barcelona was found infected. On March the 10th schools were closed in some areas of the country and, later on, on March the 14th, a partial lockdown was declared with people invited to leave their homes for work and real needs only. A national lockdown was finally declared on March the 15th. On that day almost 8 thousand of COVID-19 cases were recorded in the country (Figure 6, bottom panel).

##### 4.1.2.5. United Kingdom

**Figure 7:**
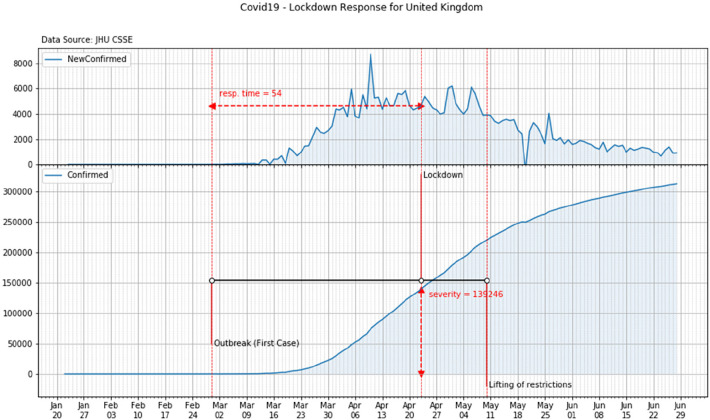
Response time and severity of the lockdown in United Kingdom.

On the 29th of February the first COVID case was detected in York, UK when two nationals were found infected. After the first death (March 6th), UK moves from the contain phase to the delay phase (March 12th) and the day after prohibition of mass gathering was banned for the next week. In the next 5 weeks, other restrictions were imposed to non-essential travels as well as schools, pubs and restaurants were closed. Between March 22nd and April 9th, the Prime Minster, the Secretary of State for Foreign Affairs, and the Queen warned they could have to introduce tougher measures and calls for self-discipline. Finally on April 23rd, the national lockdown was imposed. On that day over 139 thousand COVID-19 cases were already recorded in the country (Figure 7).

#### 4.1.3. Overall results

**Figure 8:**
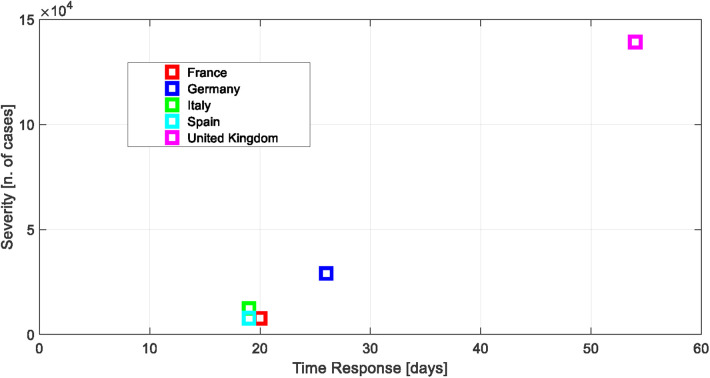
Time response vs. severity.

According to the above analysis, the overall values of the time response and of the severity of each country can be reported. These values are shown in Table 3 and in Figure 8.

**Table 3:** Time response and severity of the lockdown.

Country	Time response [day]	Severity [confirmed cases x 1000]
France	20	7.7
Germany	26	29.0
Italy	19	12.5
Spain	19	7.8
United Kingdom	54	139.3

### 4.2. Coherence of lockdown policies and mobility data

In this paragraph we aim at measuring the coherence between the lockdown policies and the people behavior in terms of their changes, if any, of their mobility

#### 4.2.1. Objective and methodology

Here we report the timeline of the national mobility of the different countries together with the date estimation of the beginning of the lockdown and of the end or steady state as it was defined in par. 3.3. For brevity, the time patterns are reported for two countries only (all other countries’ patterns are reported in the Supplementary material).

#### 4.2.2. Synthesis of results

**Figure 9:**
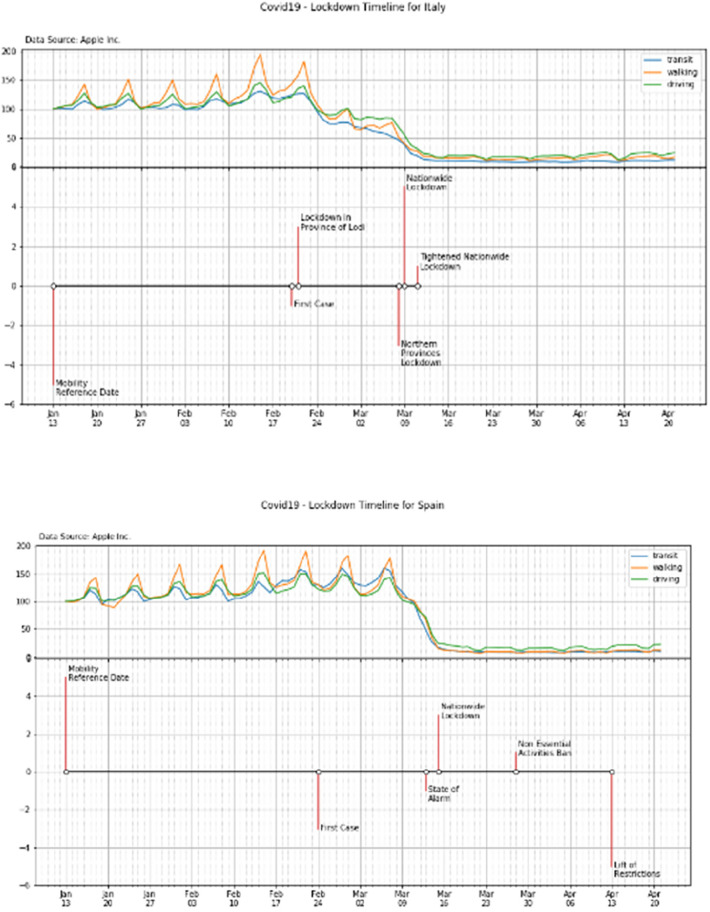
Timeline of the national mobility of Italy and Spain on the top and bottom panels, respectively.

It is important to notice that, according to a preliminary analysis of the mobility patterns, a reduction of both the Apple and Google mobilities have been observed before the official declaration of the national lockdown at least in some countries such as, for example, Italy and Spain (see Figure 9). Such behavior may have been also conditioned by the news following the identification of the fist cases in the country (see for example the mobility pattern of Italy in February after the 1st case identification—Figure 9, top panel).

Similar behaviors have also been observed in the mobility graphs of the other countries (see the Supplementary materials).

### 4.3. Effectiveness and efficiency in social distancing

Finally, we made an attempt to identify some key lockdown performance parameters in order to establish (i) responsiveness, (ii) efficiency and (iii) effectiveness of the lockdown measures

#### 4.3.1. Objective and methodology

In a second subplot we also report the derivative of the *A_m_* curves which determine the calculation of the lockdown beginning and end points (*L_i_*, *L_f_*), according to the methodology reported in par. 3. Finally, the design and plot of the two boundaries dates allow the calculation of the lockdown efficiency (Δ*T_lock_*) and effectiveness (Δ*M_lock_*) as reported below.

##### 4.3.1.1. Efficiency and effectiveness evaluation method

In order to estimate the lockdown performance and compare the timeline of the different countries, a set of performance parameters are defined as it follows

• lockdown velocity—the derivative of the 7-days rolling average of the mobility trend, namely the *S_m_*
• the beginning of the lockdown (*L_i_*)
• the steady state of the lockdown (end of lockdown for simplicity—*L_f_*)

According to the definition, efficacy and efficiency of the lockdown are then defined as it follows:

• *efficiency*—the time that is spent between the beginning and end of the lockdown, namely

$$\Delta T_{lock}=T_{f}-T_{i}$$

where Δ*T_lock_* is the efficiency of the lockdown, *T_f_* and *T_i_* are the *L_f_* and *L_i_* dates, respectively.

• *effectiveness*—the observed drop of the mobility drop due to the lockdown between *T_i_* and *T_f_*, namely

$$\Delta M_{lock}=M_{f}-M_{i}$$

where Δ*M_lock_* is the effectiveness of the lockdown, *M_f_* and *M_i_* are the values of the mobility at the time (or dates) *L_f_* and *L_i_*, respectively.

**Figure 10:**
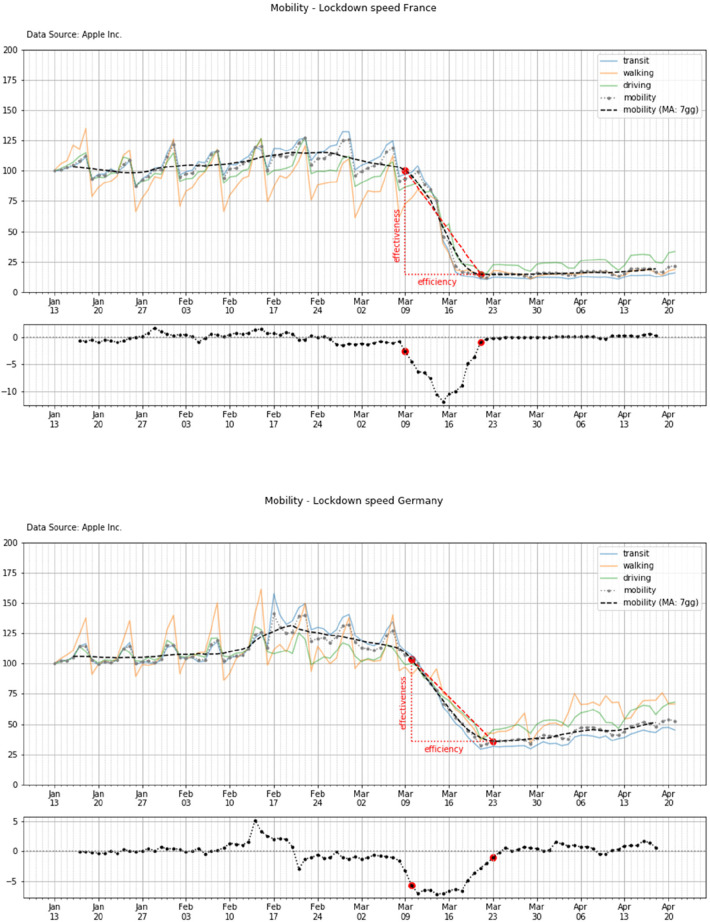
Lockdown and mobility timeline in France and Germany (top and bottom panels, respectively).

Figure 10 (top and bottom panels) shows an example of the calculation of such efficiency and efficacy for two countries (i.e., France and Germany). Similar plots and calculations can be performed for the other countries.

#### 4.3.2. Outcome by country

Here we report the lockdown and mobility timeline of the different countries. For brevity, the time patterns are reported for two countries only (all other countries' patterns are reported in the Supplementary material).

#### 4.3.3. Synthesis of results

Table 4 shows the value of the lockdown efficiency and efficacy for each of the analyzed countries. At this stage of the analysis it is important to observe that all countries show a significant reduction of the population mobility (i.e., the effectiveness—mean and std: 78.6 ± 6.5), even if the efficiency of such a reduction is significantly different with a very high standard deviation (mean and std: 14.6 ± 4.7).

**Table 4:** Efficiency and effectiveness of each country lockdown.

Country	Lockdown performance			
	*L_i_*	*L_f_*	Δ*T_lock_* Efficiency [days]	Δ*M_lock_* Effectiveness [%]
*France*	09/03	21/03	12	85
*Germany*	10/03	23/03	13	70
*Italy*	24/02	15/03	20	85
*Spain*	10/03	19/03	9	78
*United Kingdom*	09/03	28/03	19	75

In particular, countries such as Italy and United Kingdom show a tendency to require many days in order to perform an effective lockdown. Such a slow speed of the lockdown execution may have had a strongly impact on the spread of COVID-19.

### 4.4. Effects of social distancing on the epidemic evolution

Finally, we made an attempt *to* model the latency occurring between the changes in social behaviors and the changes in growth rate of the disease.

#### 4.4.1. Objective and methodology

Here we combine the mobility timeline with the temporal pattern of the infection: three curves are plotted; these curves represent

(i) the mobility patterns
(ii) the number of *NewConfirmed* (definition in par. 3.2)
(iii) a semi-transparent time shifted curve of the mobility

The time shift of this curve is arbitrary and it is performed in order to align the descending pattern of the mobility with the descending pattern of the infection. The purpose of such a strategy is to highlight and qualitative determine the effective time shift (Δ*S*) which is needed to see a benefit of the lockdown vs. the reduction of the infection.

#### 4.4.2. Outcome by country

**Figure 11:**
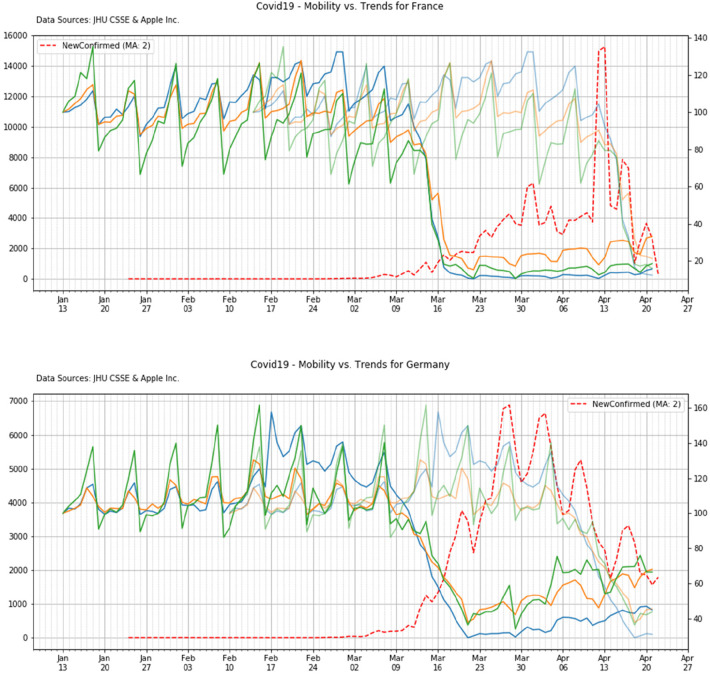
Mobility vs. COVID-19 trends of United Kingdom.

Here we have reported the main timeline events of the lockdown policy for two countries --namely France and Germany'vs. the time pattern of the new confirmed cases (Figure 11).

#### 4.4.3. Synthesis of results

In Table 5 we reported a the amount of virtual time shift which is needed in order to align the lockdown with the effective reduction of the new confirmed cases. It is important to observe that, irrespective of the initial condition of the outbreak for each country (i.e., the initial number of cases at the beginning of the lockdown) the shift time is always in the range between 27 and 35 days (mean and std: 31.6 ± 4.0), therefore a period of at least 3–4 weeks is needed in every country in order to see some initial benefit of the social distance policies.

**Table 5:** Time shift for the alignment of the decreasing trends of the outbreak vs. the lockdown.

Country	Δ*S* time shift [days]
*France*	32
*Germany*	28
*Italy*	35
*Spain*	27
*United Kingdom*	36

## 5. Discussion and next steps

The analysis was conducted on the following 5 countries in Europe (France, Germany, Italy, Spain, United Kingdom).

These countries were chosen because of their similarities from a geographical and cultural viewpoint: that makes a comparison easier. Moreover, all of them experienced the COVID or Coronavirus outbreak in the “same” period and represent a large share of the overall number of COVID-19 cases in the European continent.

First, the mobility reported from Apple, even if representing a map research task of the end-user, seems well aligned with the data which are provided by Google: the next figure shows a comparison of Apple and Google data after aligning the dates of the data from the two database for one country (i.e., Italy). Similar patterns are observed for the other countries.

In this plot we also show the time scale of the official and most significative dates of the lockdown together with the timeline of the new confirmed cases (top panel) and, more importantly, with the overall number of cases (bottom plot). This final figure is extremely important because it shows the importance of performing a proper and strict lockdown when the initial condition of the infection (i.e., the number of confirmed new cases and overall cases) is low, otherwise—even if the lockdown is efficient and effective—the spread of the infection will rapidly occur whatever social distancing policy will be applied.

**Figure 12:**
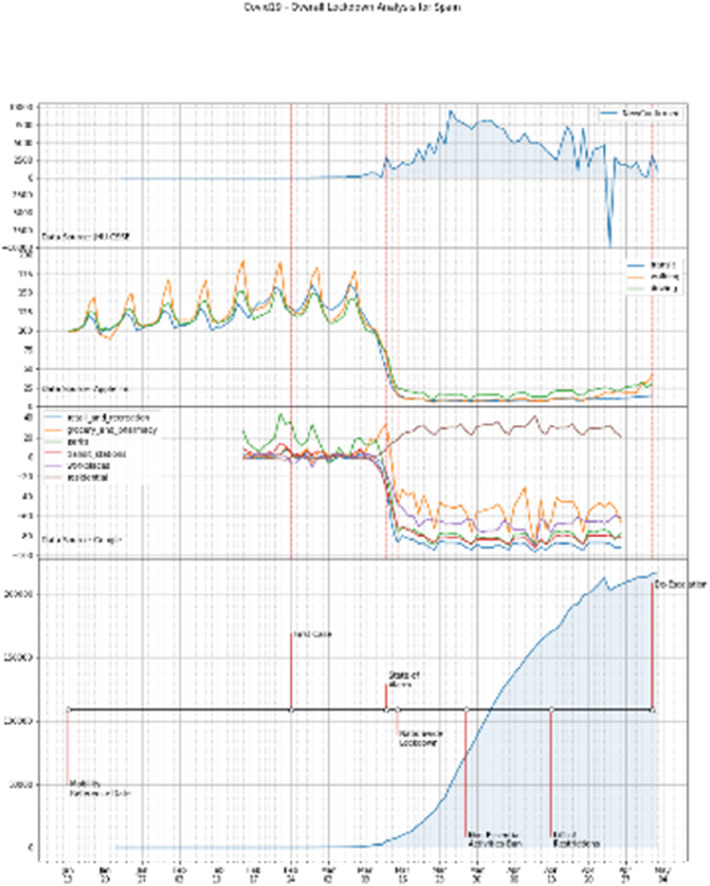
Overall lockdown analysis. Countries: Spain and Italy, top and bottom panels, respectively.

The following Figure 12 summarizes the overall lockdown analysis of two representative countries, i.e., Spain and Italy. Similar summary can be obtained from the graphs of the other countries.

## 6. Conclusions

This paper integrates a preliminary analysis of the population mobility with the COVID-19 outbreak in 5 European Countries. Worldwide Apple and Google mobility data have been processed and combined with the John Hopkins database of COVID-19 outbreak.

The Apple and Google database, as well as the JHU COVID-19 database are currently reporting data of all the countries in the world. At this stage we decided to only focus some of the most populated and larger European countries which represent a large amount of the European population. Nevertheless, it is worth mentioning that the way our software manipulates these data would allow the selection of other countries' data to be analyzed, and therefore an extension to other countries and areas would be relatively easily performed.

We defined a set of parameters for determining the beginning and steady state of each country lockdown, as well as a set of performance parameter to estimate the performance of the lockdown. For each country these parameters have been calculated vs. the behavior of the COVID-19 infection, i.e., the confirmed and cumulative number of cases of each country.

In this framework we also look at the number of weeks or period of time and shift that is needed in order to see an effective reduction of the virus in the population *after* the lockdown.

We have also analyzed the influence of the initial condition of the outbreak (i.e., the effective number of cases at the beginning of the lockdown) with respect to the timeline of the contagious occurring after the lockdown.

This is clearly a preliminary study and further work should be developed in order to deeply analyze the population behavior at national and sub-national/regional level, as well as, to look more specifically at the effect of the lockdown vs. a more detailed model of the COVID-19 diffusion (3, 8).

In this context, it is worth to mention how important would be to embed into this analysis the roles of the hospitalization of those members of the population that are requiring it, as well as the main role of the quarantine measure (5). Another very critical aspect, which has not been included in this analysis, despite its importance, is the effect of single or multiple vaccine injections vs. the spread of the virus and, as a consequence, vs. the lockdown strategies: this particular aspect would deserve a specific study (4, 6, 9).

Finally, it is important to underline that this study is not exhaustive, rather should inspire further research aimed at supporting the development of epidemiologist modelling where, for example, the distribution of the hospitals and of poles of attraction of people would be taken into account, together with an evaluation of the lockdown impact vs. the outbreak (2, 7, 22). For example, the data analysis could be clustered around these poles of attractions (i.e., “retail and recreation”, “workplaces”, etc.) and crossed with the National Health data (i.e., status of the vaccination, number of contacts, etc.).

The Supplementary Material Section (i.e., Section 8) reports all the figures and plots for each of the analyzed country. Details of the software are available on request.

## Data Availability

The original contributions presented in the study are included in the article/**Supplementary Material**, further inquiries can be directed to the corresponding author/s.

## Footnotes

^1^Python.org. 2020. Welcome To Python.Org. [online] Available at: https://www.python.org/ [Accessed 7 May 2020].

^2^Jupyter.org. 2020. Project Jupyter. [online] Available at: https://jupyter.org/ [Accessed 7 May 2020].
