# Supplementary material for: To lockdown or not to lockdown: Analysis of the EU lockdown performance vs. COVID-19 outbreak
Source: Front Med Technol. 2022 Oct 21;4:981620. doi: 10.3389/fmedt.2022.981620 (PMC9634555; doi:10.3389/fmedt.2022.981620)
Supplement: Supplementary file 1 [file Datasheet1.pdf]

Supplementary Material

Contents

8 Supplementary Data 1
8.1 Paragraph 2.1.3 2
8.2 Paragraph 2.2.3 5
8.3 Paragraph 3.2 8
8.4 Paragraph 3.3.1 10
8.5 Paragraph 3.3.1 13
8.6 Paragraph 3.3.2 16

8 Supplementary Data

Here we report the plot of all countries, given that the paragraphs of the article usually report the plots of few countries only.

8.1 Paragraph 2.1.3

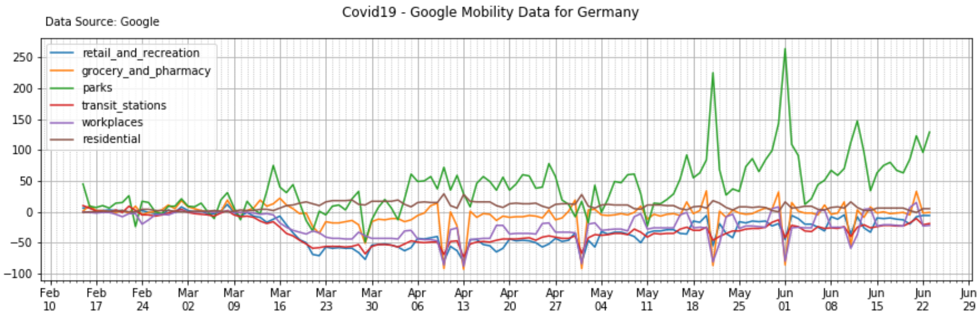

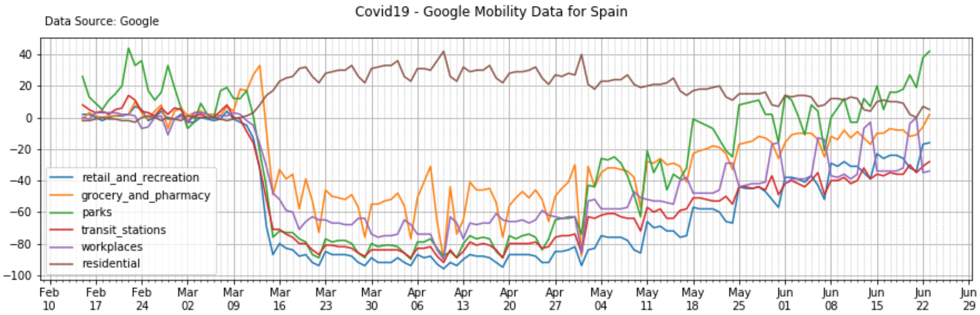

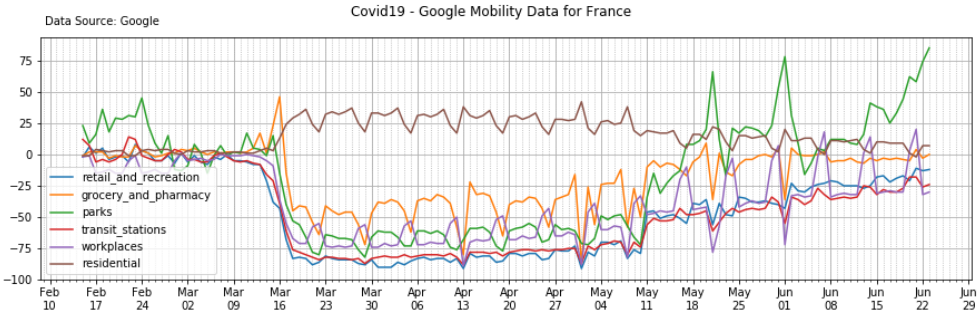

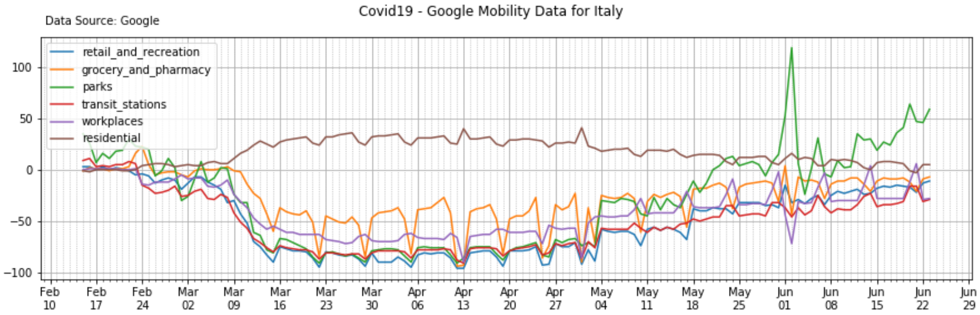

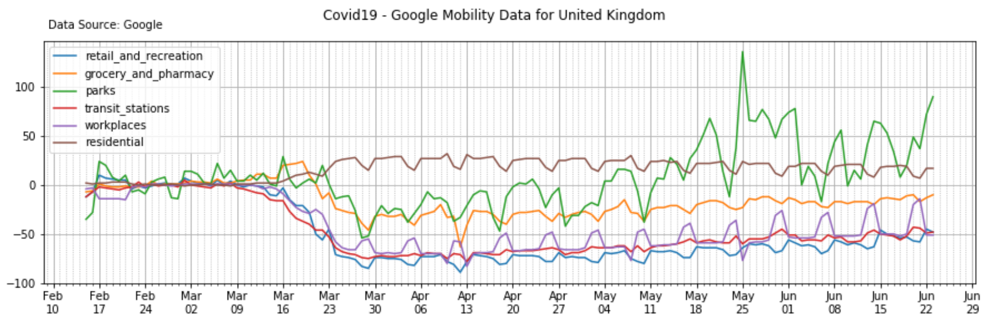

Figure 1 - Google mobility trends of all the 5 countries, from the top to the bottom panel, Germany, Spain, France, Italy, United Kingdom, respectively.

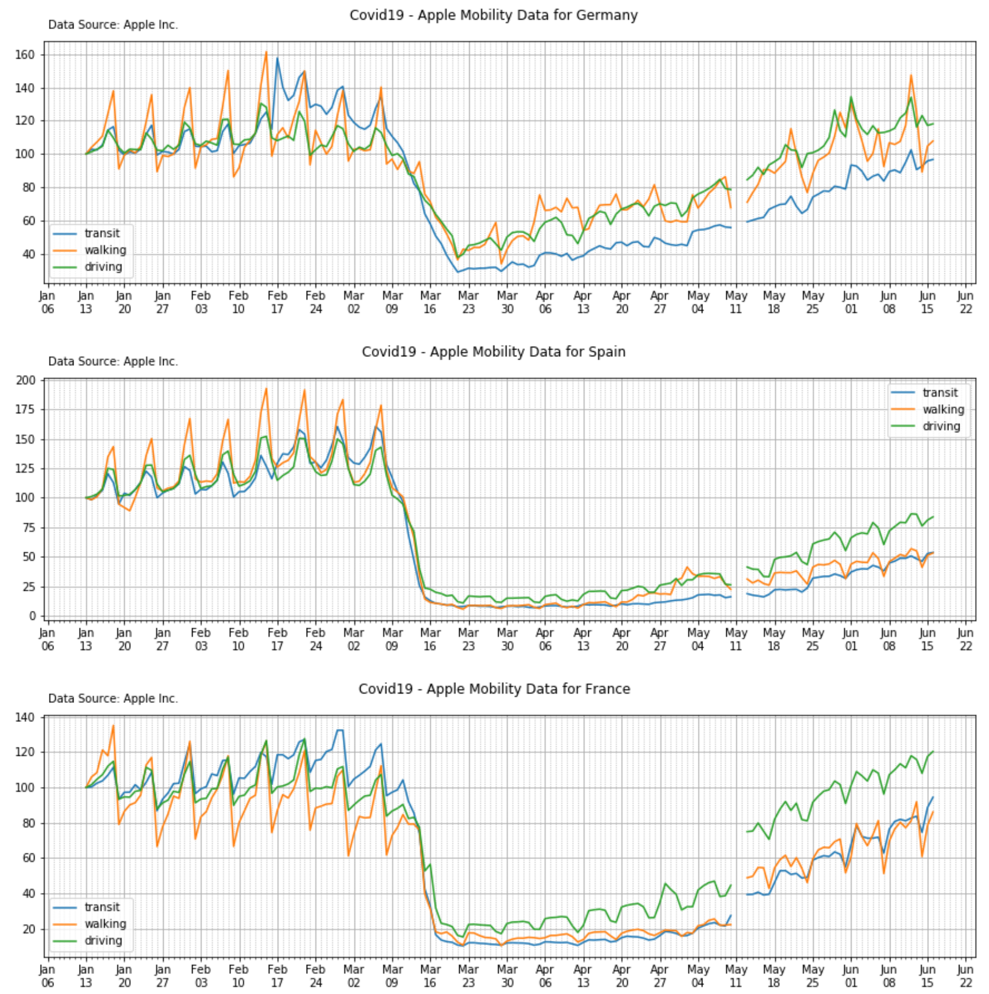

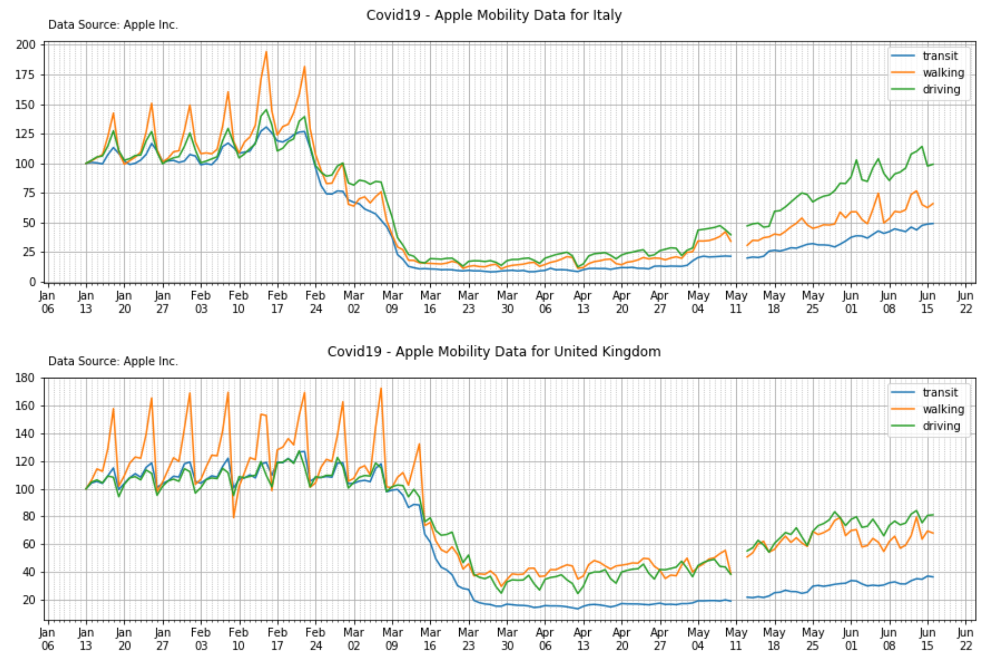

Figure 2 – Apple mobility trends of all the 5 countries, from the top to the bottom panel, Germany, Spain, France, Italy, United Kingdom, respectively.

8.2 Paragraph 2.2.3

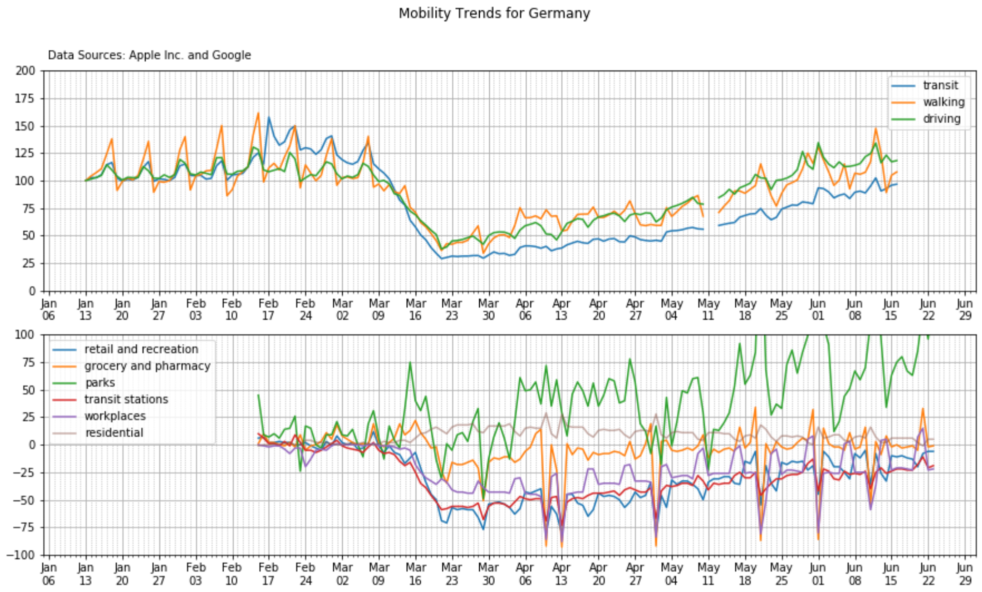

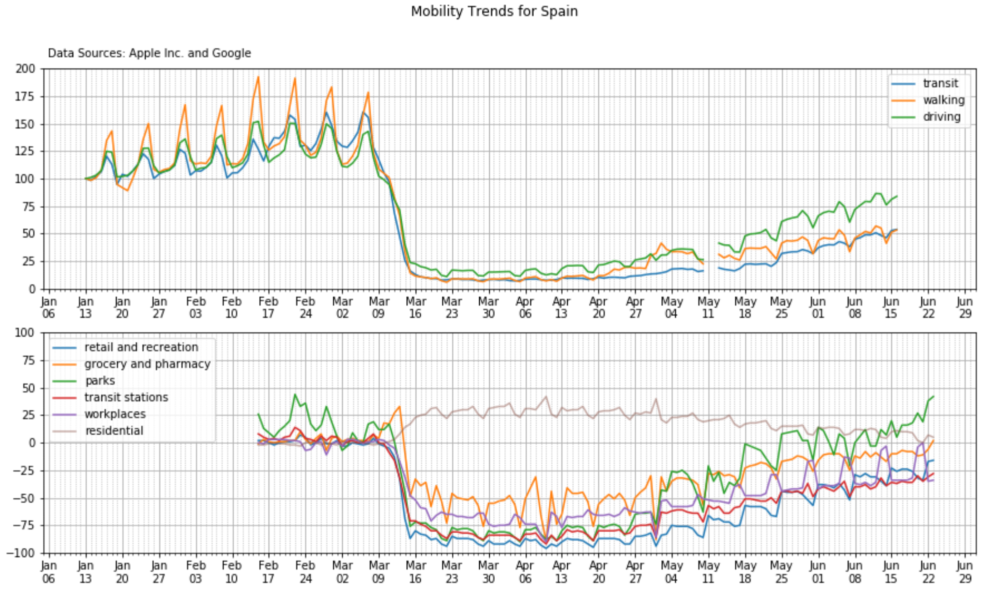

Mobility Trends for France

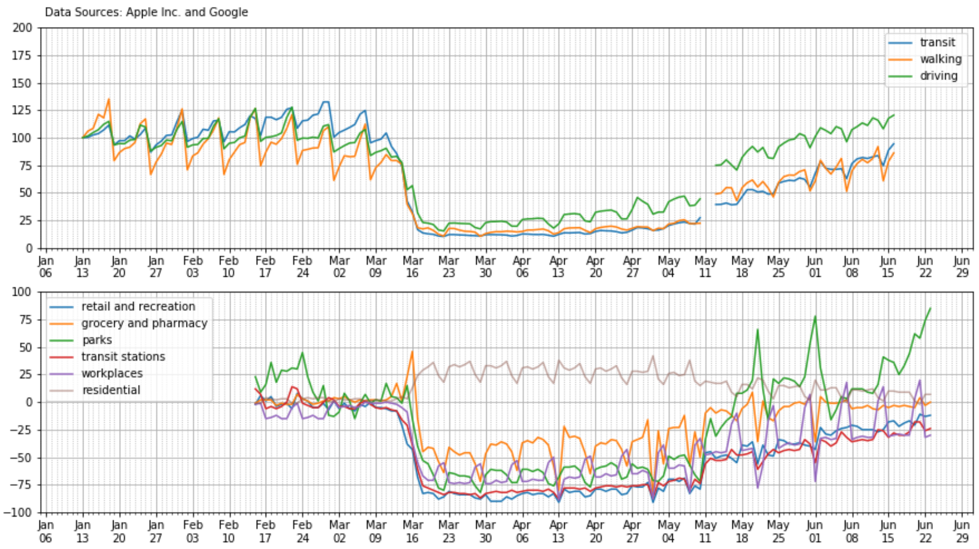

Mobility Trends for Italy

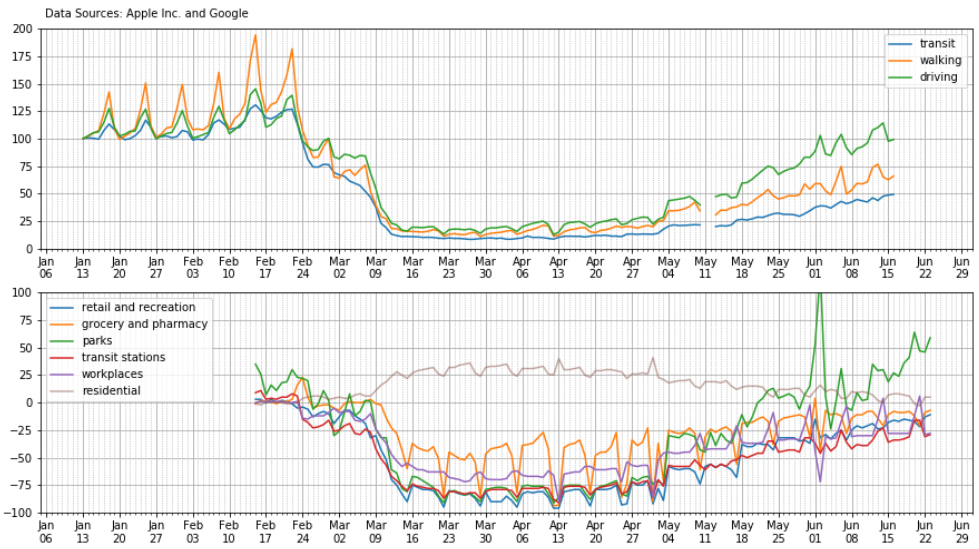

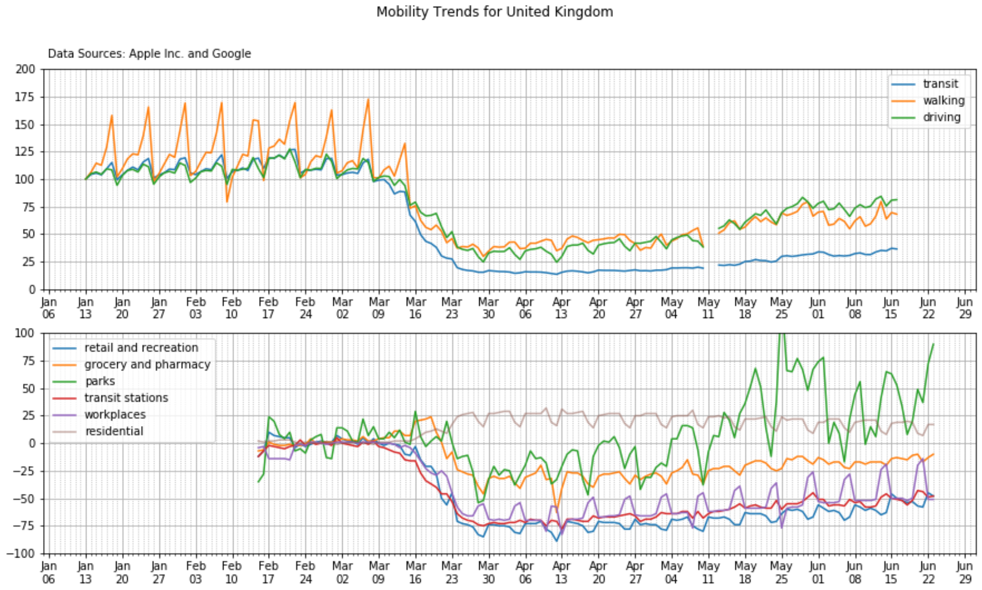

Figure 3 - time alignment of the Apple and Google data - top and bottom sub-panels respectively - of all the 5 countries, from the top to the bottom panel, Germany, Spain, France, Italy, United Kingdom, respectively

8.3 Paragraph 3.2

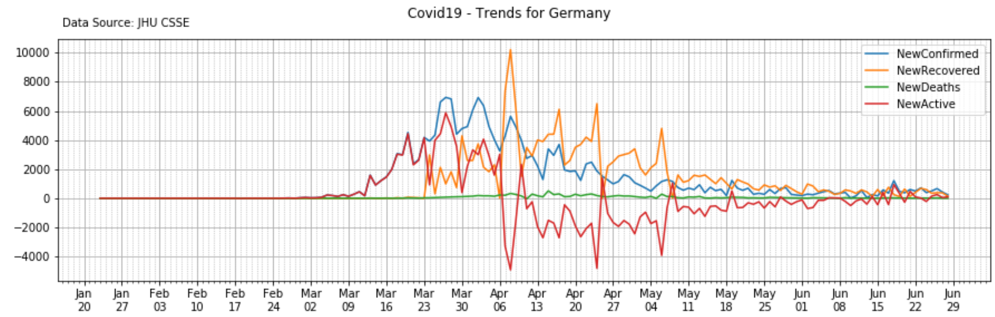

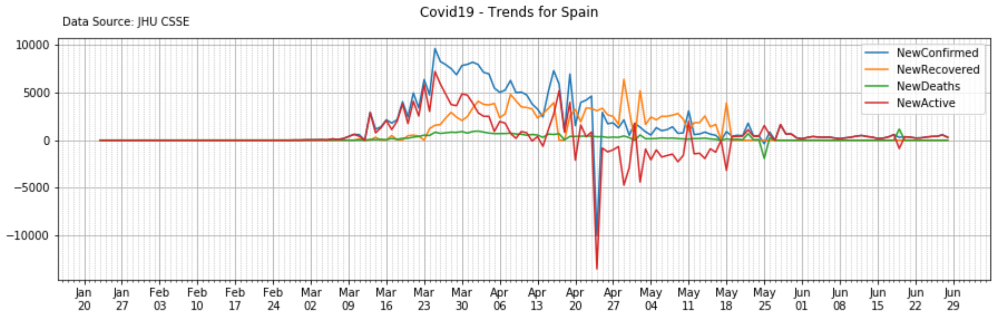

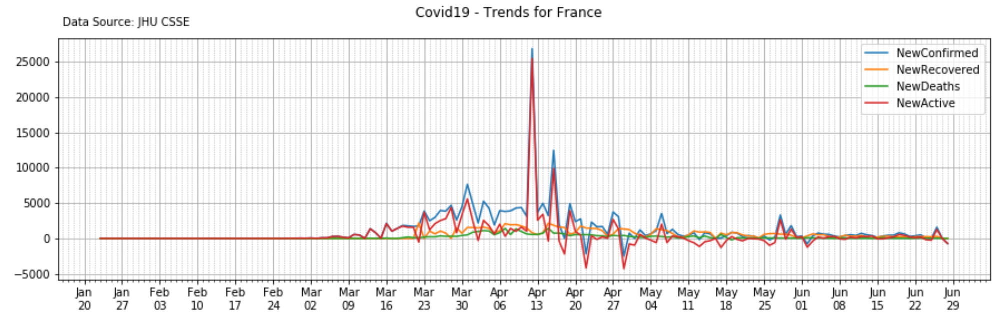

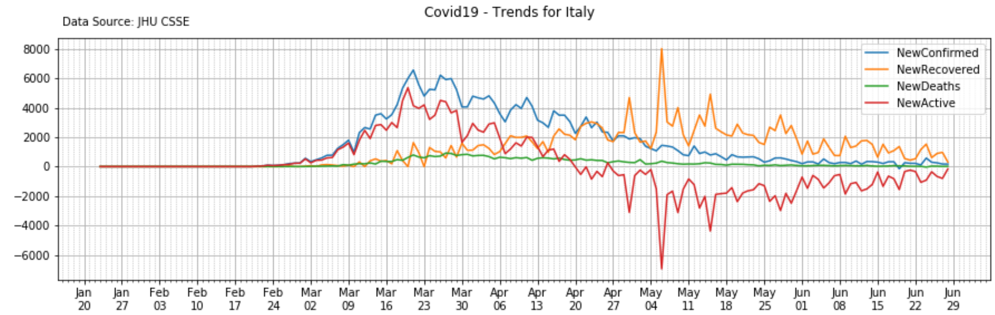

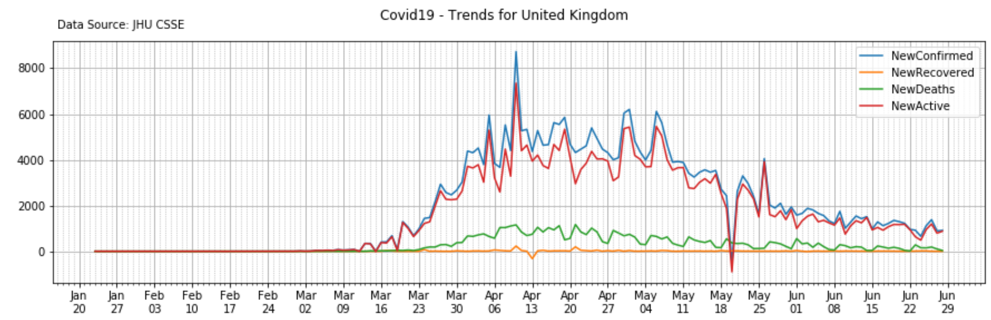

Figure 4 - NewConfirmed, NewActive, NewRecoverd and NeawDeaths Trends of all the 5 countries, from the top to the bottom panel, Germany, Spain, France, Italy, United Kingdom, respectively.

8.4 Paragraph 3.3.1

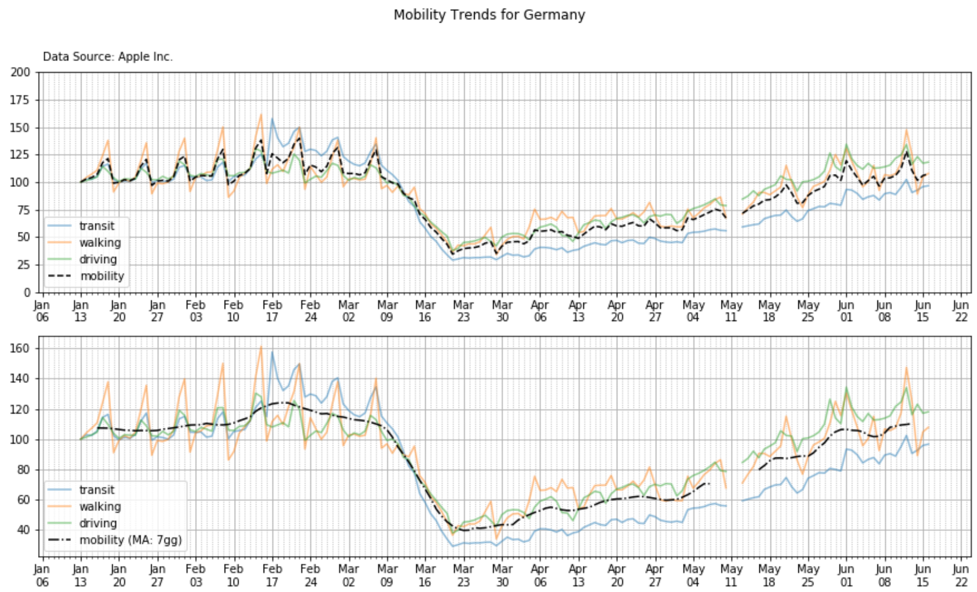

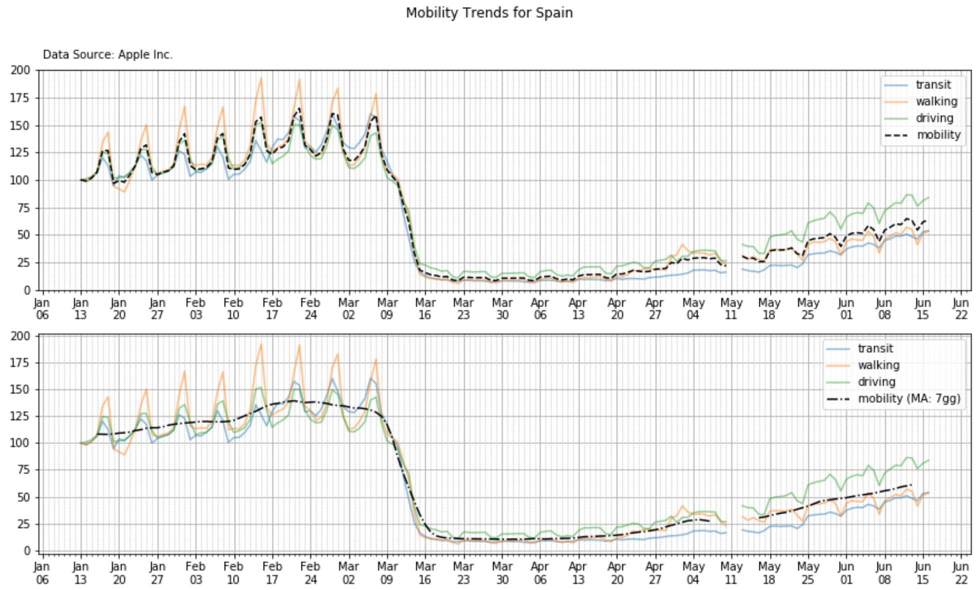

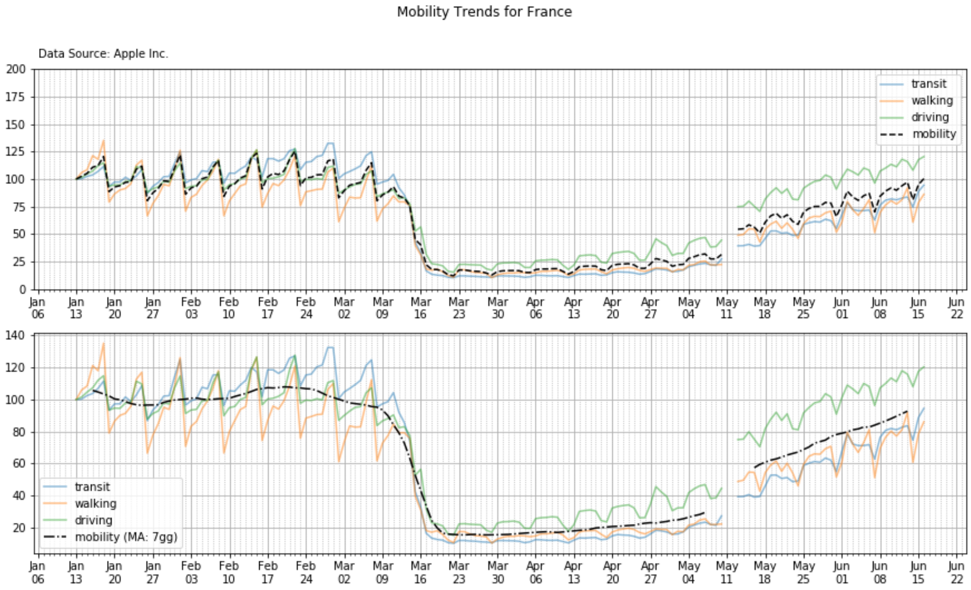
Mobility Trends for France
Data Source: Apple Inc.
transit
walking
driving
mobility
mobility (MA: 7gg)

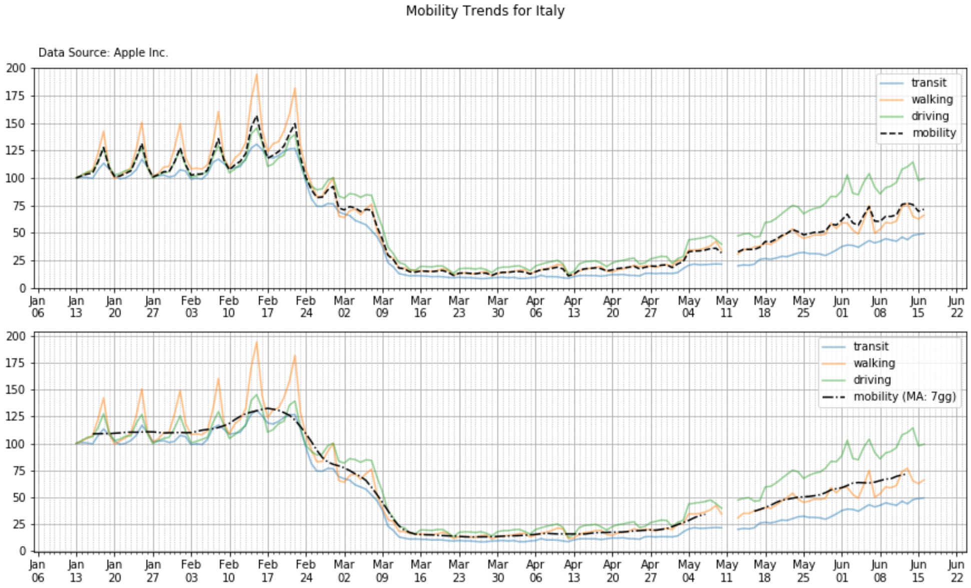
Mobility Trends for Italy
Data Source: Apple Inc.
transit
walking
driving
mobility
mobility (MA: 7gg)

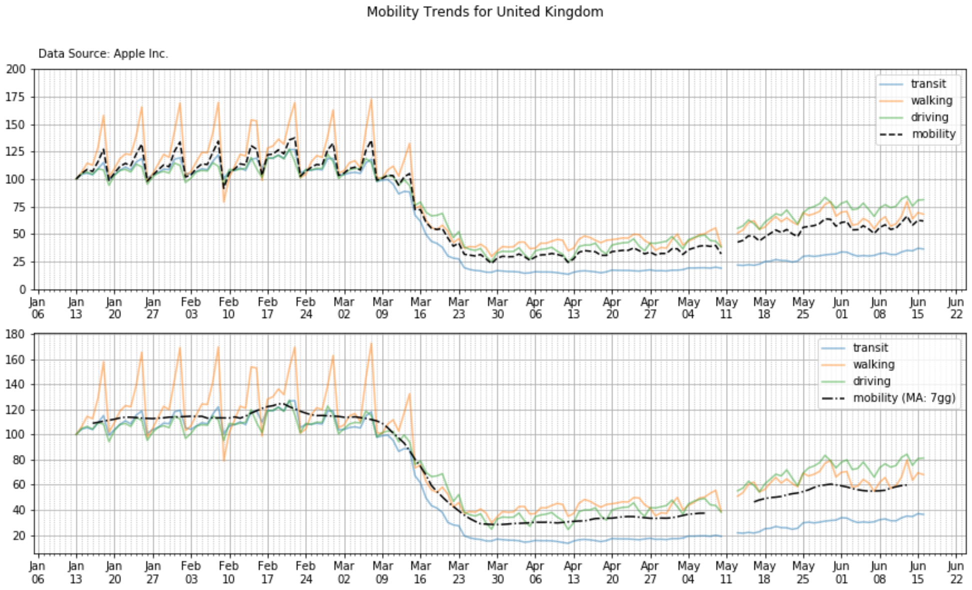

Figure 5 - Average (top panel) and 7-days rolling average (bottom panel) of the Apple Mobility data of all the 5 countries, from the top to the bottom panel, Germany, Spain, France, Italy, United Kingdom, respectively.

8.5 Paragraph 3.3.1

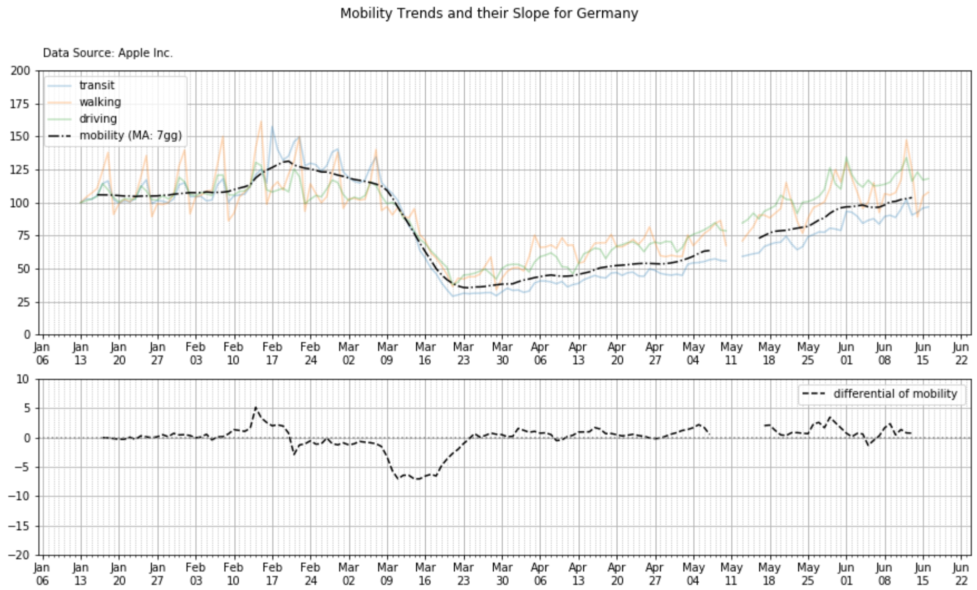

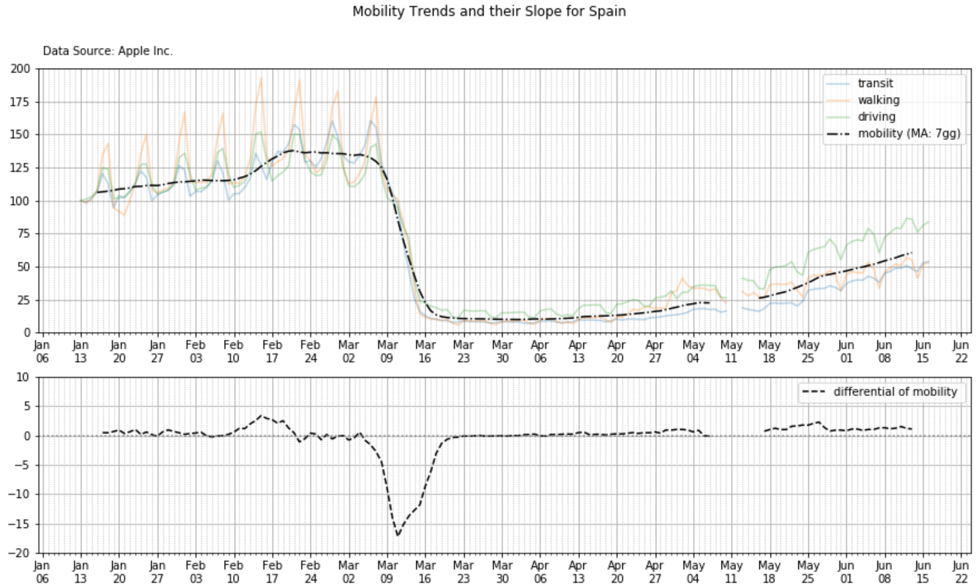

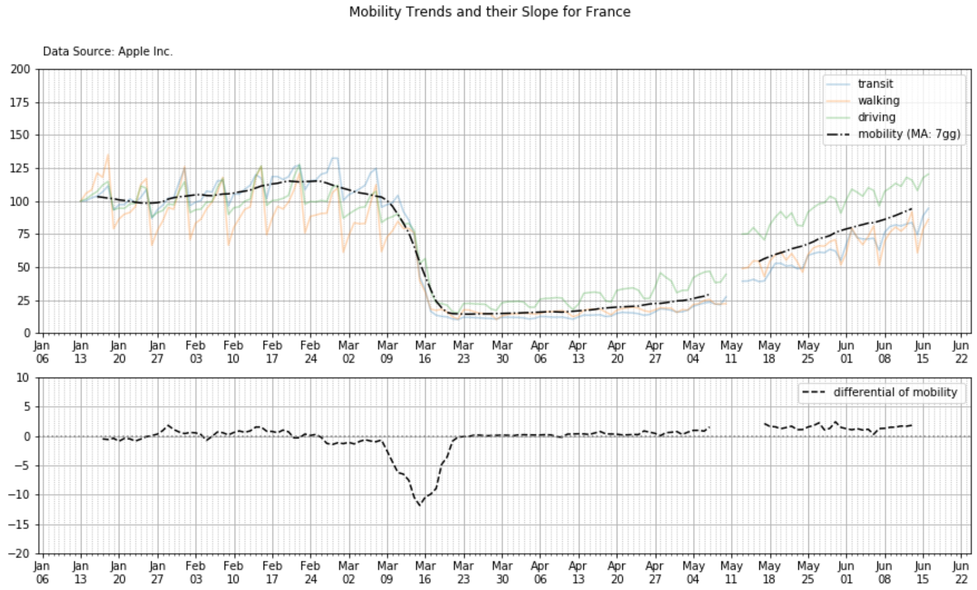
Mobility Trends and their Slope for France
Data Source: Apple Inc.
transit
walking
driving
mobility (MA: 7gg)
differential of mobility

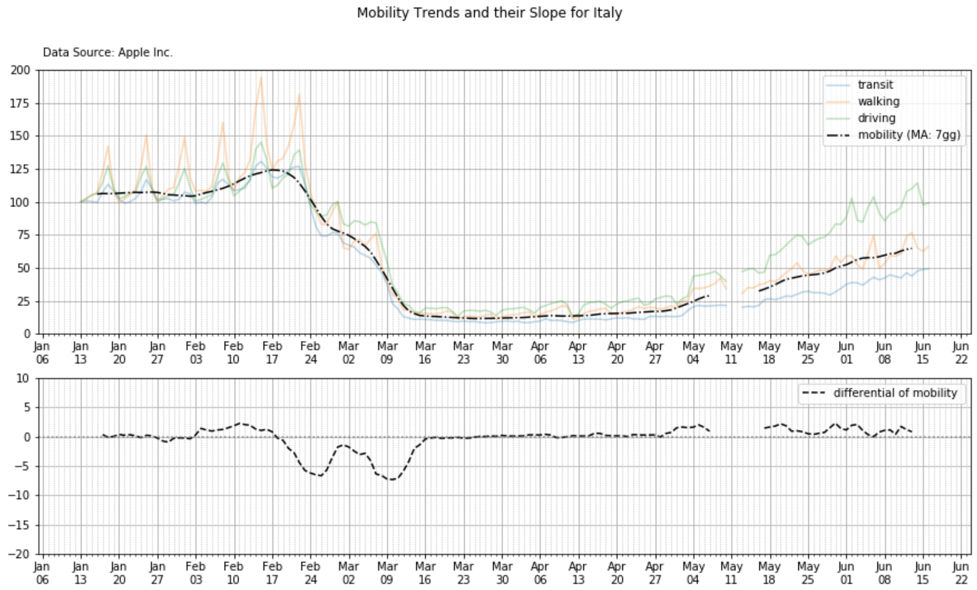
Mobility Trends and their Slope for Italy
Data Source: Apple Inc.
transit
walking
driving
mobility (MA: 7gg)
differential of mobility

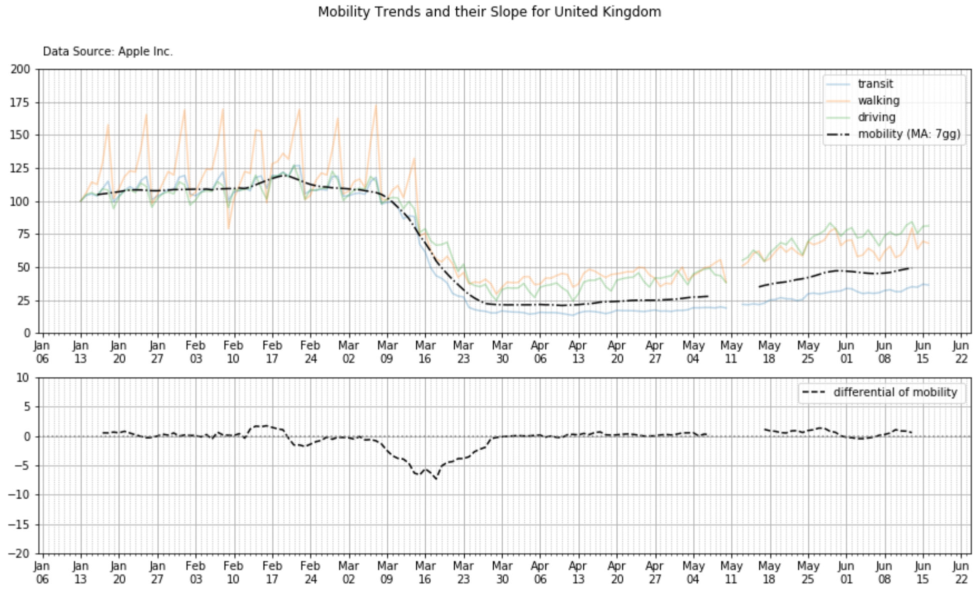

Figure 6 - Top sub-panel - The 7-days rolling average mobility as extracted from the original data set provided by Apple: the residential mobility trends are averaged and then smoothed (black dotted line). Bottom sub-panel – the derivative of the 7-days rolling average curve. Countries: from top to bottom, Germany, Spain, France, Italy, United Kingdom, respectively.

8.6 Paragraph 3.3.2

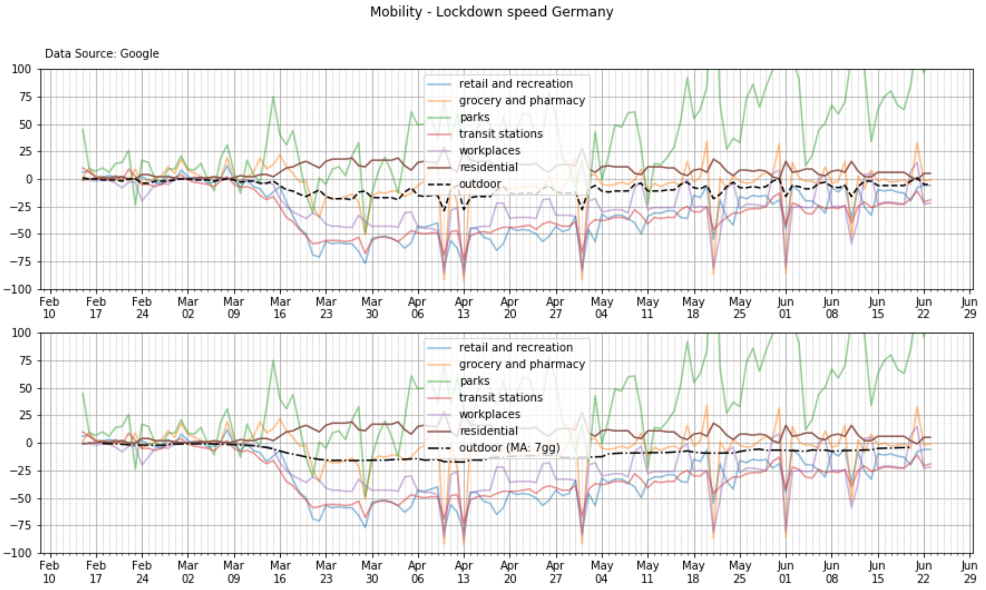

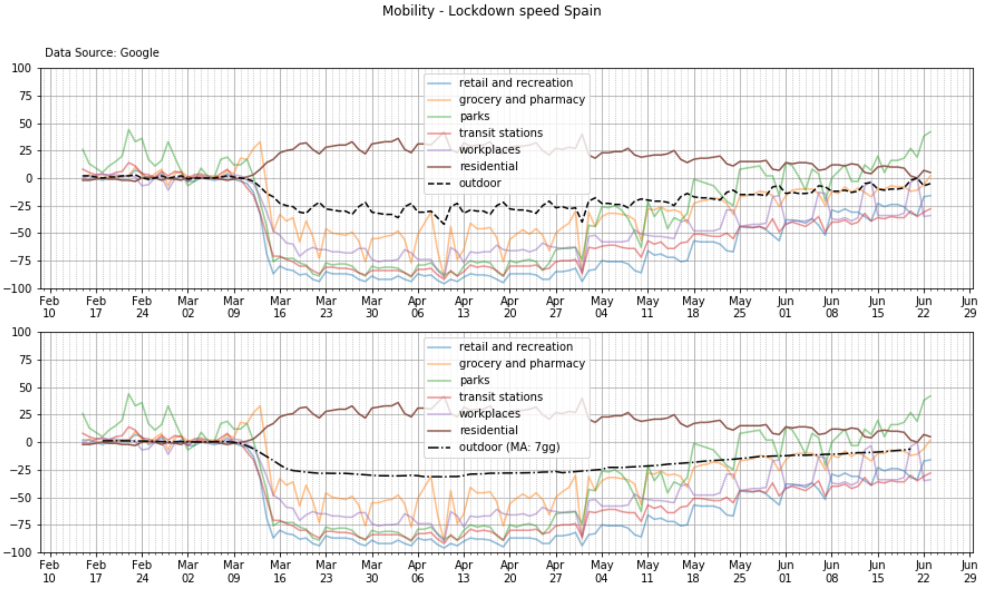

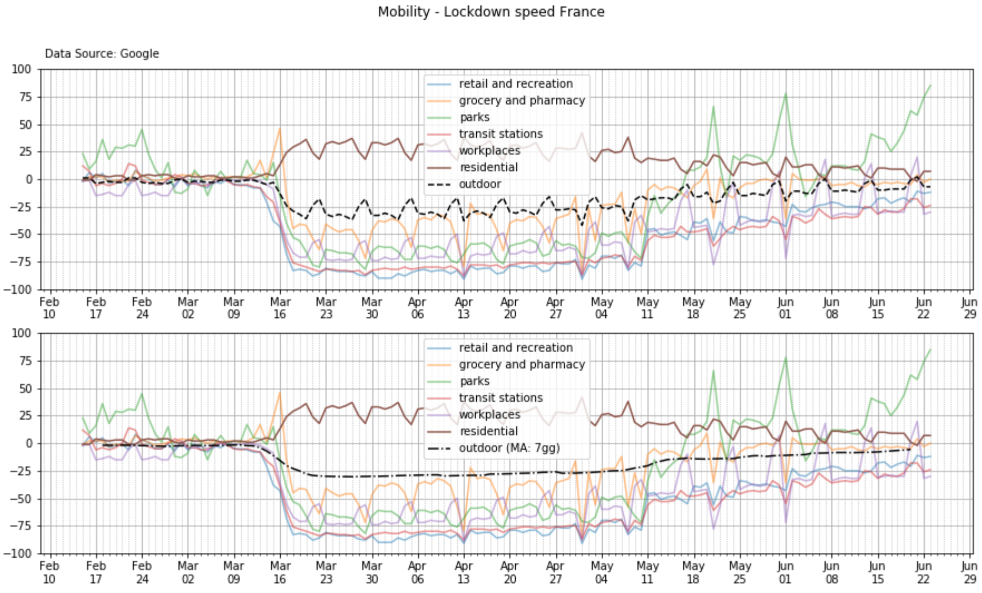
Mobility - Lockdown speed France
Data Source: Google
retail and recreation
grocery and pharmacy
parks
transit stations
workplaces
residential
outdoor
outdoor (MA: 7gg)

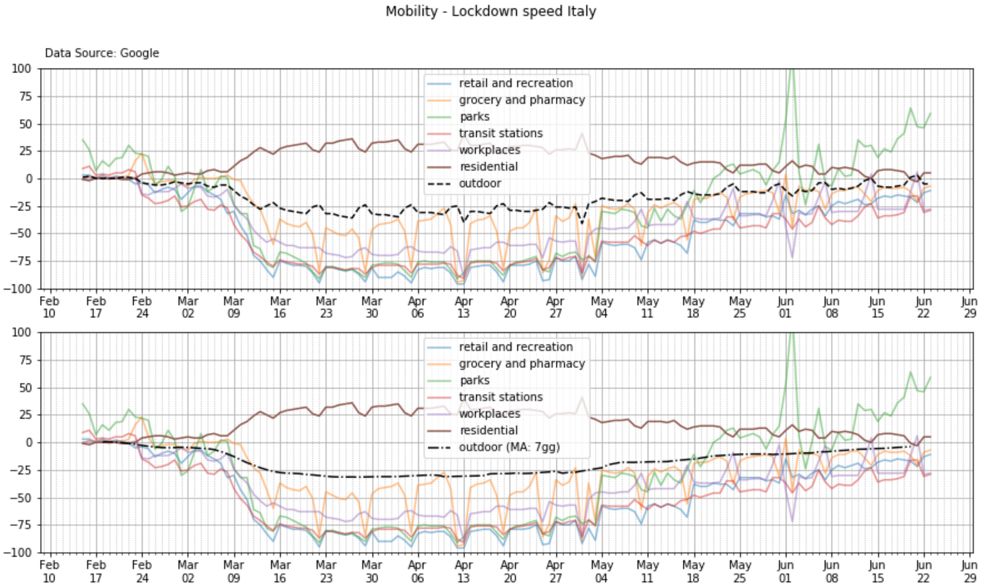
Mobility - Lockdown speed Italy
Data Source: Google
retail and recreation
grocery and pharmacy
parks
transit stations
workplaces
residential
outdoor
outdoor (MA: 7gg)

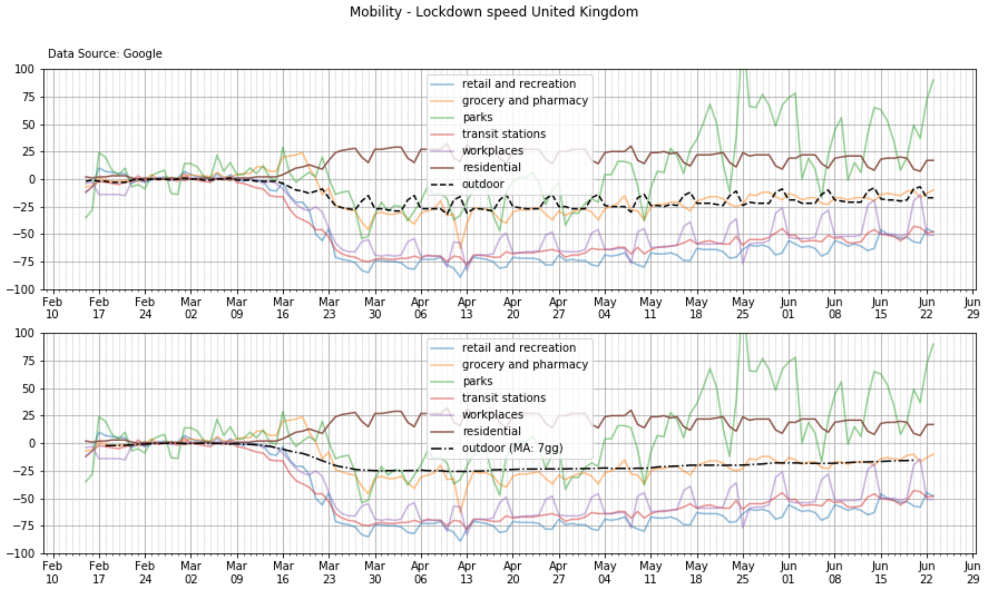

Figure 7 - Average (top sub-panel) and 7-days rolling average (bottom sub-panel) of original and reversed Google Mobility data (top and bottom sub-panels, respectively) - see details in the text for the definition of reversed data. Countries: from top to bottom, Germany, Spain, France, Italy, United Kingdom, respectively.

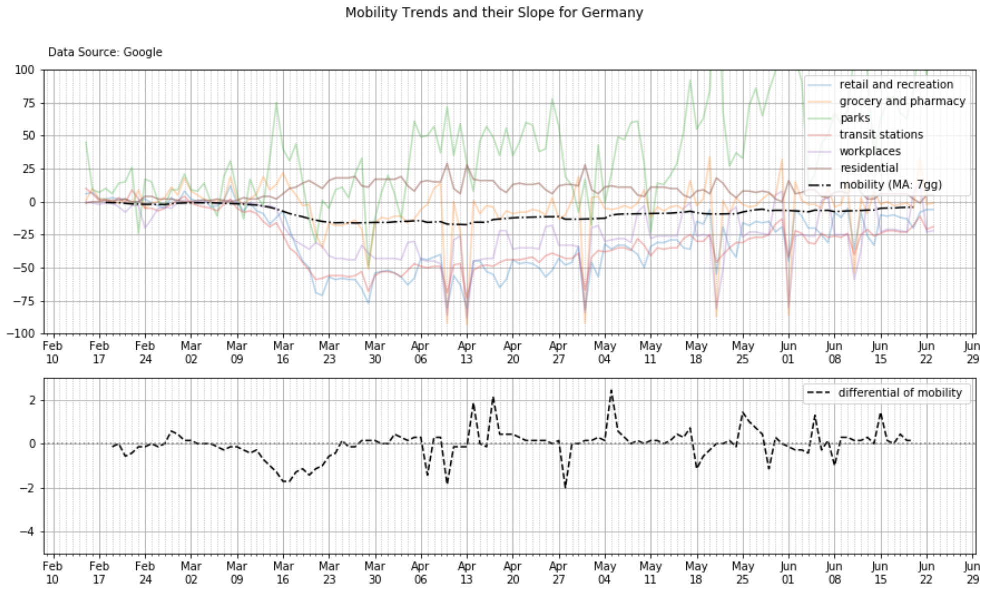

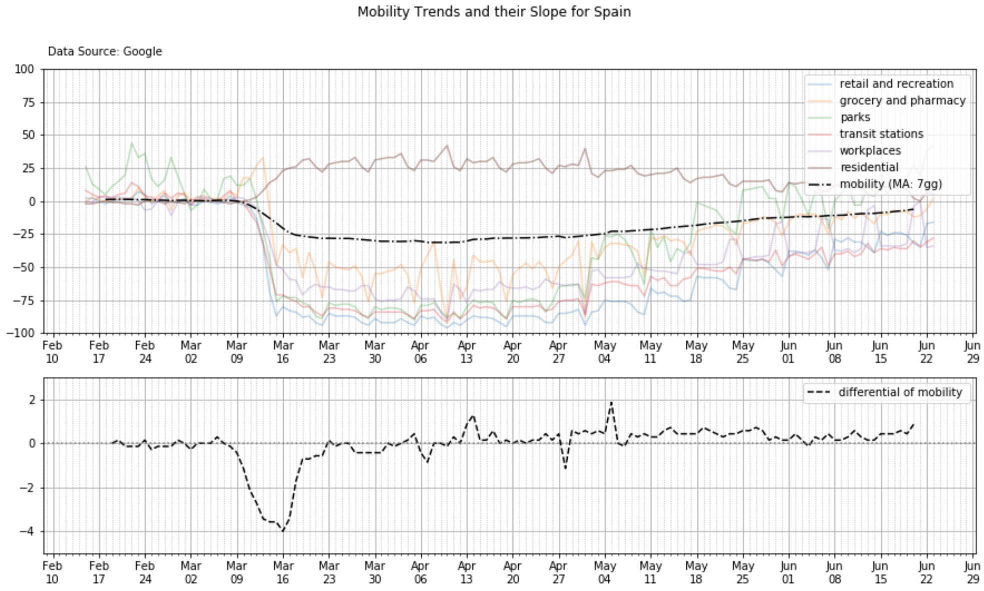
Mobility Trends and their Slope for Spain
Data Source: Google
retail and recreation
grocery and pharmacy
parks
transit stations
workplaces
residential
mobility (MA: 7gg)
differential of mobility

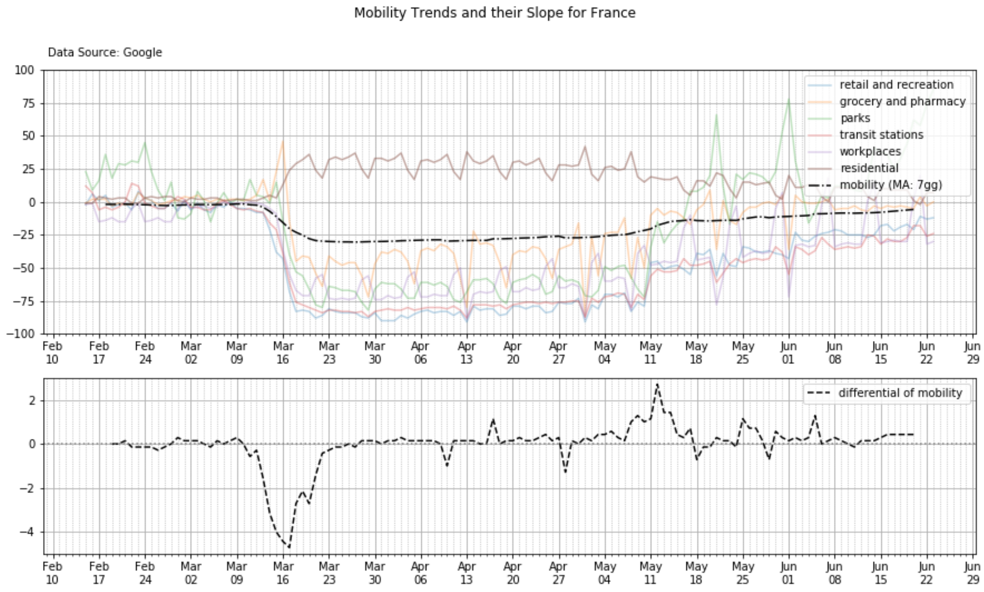
Mobility Trends and their Slope for France
Data Source: Google
retail and recreation
grocery and pharmacy
parks
transit stations
workplaces
residential
mobility (MA: 7gg)
differential of mobility

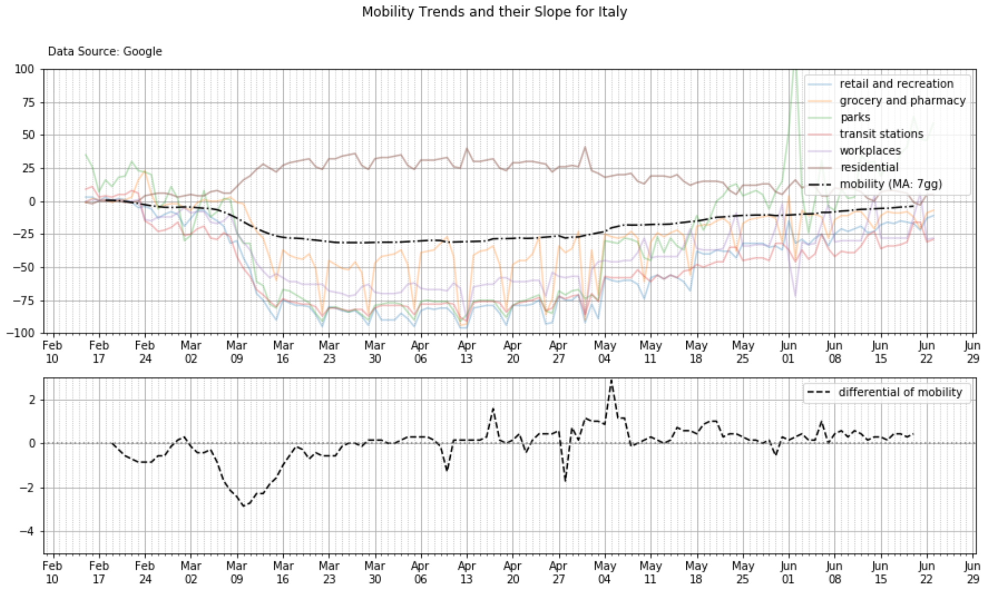

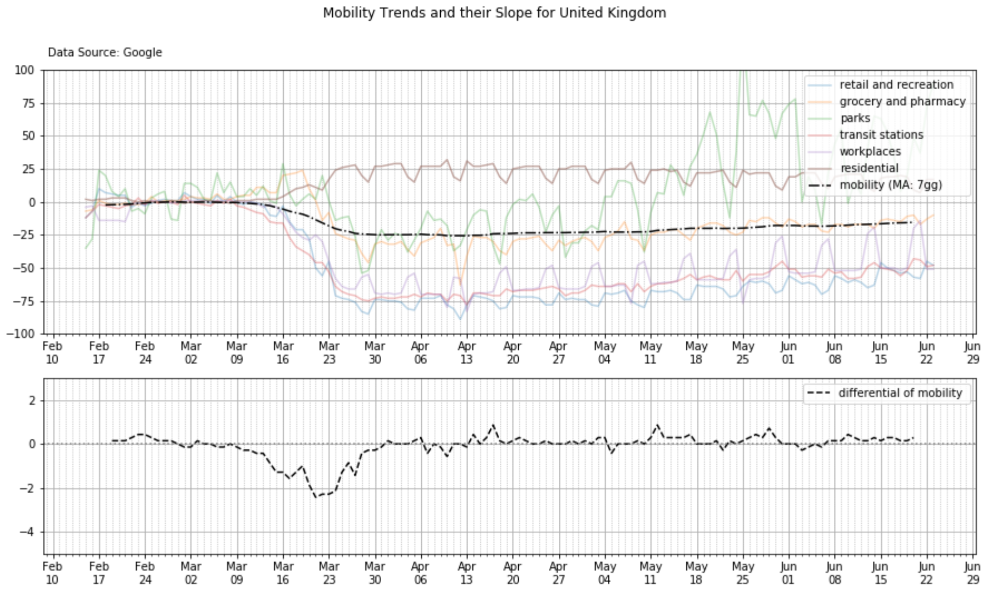

Figure 8 - Top sub-panel - The 7-days rolling average mobility as extracted from the original data set provided by Google: the residential mobility trends is reversed and then smoothed (black dotted line). Bottom sub-panel – the derivative of the 7-days rolling average curve. Countries: from top to bottom, Germany, Spain, France, Italy, United Kingdom, respectively.
